# Type 2 immune history imprints local training in nerve and airway associated interstitial macrophages (NAMs) for disease tolerance during lethal respiratory viral infection

**DOI:** 10.21203/rs.3.rs-8780633/v1

**Published:** 2026-02-12

**Authors:** Payal Damani-Yokota, Yavor Yordanov, Eduardo D. Bernier, Chaitra Sreenivasaiah, Alireza Khodadadi-Jamayran, Matthias Kugler, Stephen T. Yeung, Stacey Bartlett, Valeria Mezzano, Eric Bartnicki, Mingjun Liu, Fei Chen, William C. Gause, Aristotelis Tsirigos, Iannis Aifantis, Mila Ortigoza, Bettina Nadorp, Musa Mhlanga, Kamal M. Khanna

**Affiliations:** 1 Department of Microbiology, New York University Grossman School of Medicine, New York, New York, USA.; 2 Department of Internal Medicine and Radboud Center for Infectious Diseases, Radboud University Medical Center, Nijmegen, the Netherlands; 2 Department of Cell Biology, Faculty of Science, Radboud Institute for Molecular Life Sciences, Radboud University Nijmegen, Nijmegen, the Netherlands; 2 Department of Human Genetics, Radboud University Medical Center, Nijmegen, the Netherlands; 4 Applied Bioinformatics Laboratories, Office of Science and Research, New York University School of Medicine, New York, NY, USA., United States; 5 Division of Pulmonary and Critical Care Medicine, New York University, New York, NY, USA; 6 Department of Medicine/Division of Infectious Diseases and Immunology, New York University Grossman School of Medicine, New York, NY, 10016, USA; 7 Department of Pathology, NYU Grossman School of Medicine, New York, NY, USA; 8 Center for Immunity and Inflammation, New Jersey Medical School, Rutgers-The State University of New Jersey, Newark, NJ, USA; Department of Medicine, New Jersey Medical School, Rutgers-The State University of New Jersey, Newark, NJ, USA.; 9 Division of Precision Medicine, Department of Medicine, New York University Grossman School of Medicine, New York, NY; 10 Perlmutter Cancer Center, New York University Langone Health, New York, NY, USA

**Keywords:** macrophages, trained immunity, NAMs, innate immune memory, disease tolerance, efferocytosis, type 2 immunity, tissue-repair

## Abstract

Severe respiratory viral disease varies widely among individuals and often reflects immunopathology rather than inadequate pathogen control, suggesting that prior immune history can prime the lung’s inflammatory–regulatory balance to promote disease tolerance. Here we show that nerve- and airway-associated macrophages (NAMs), a subset of interstitial macrophages expand following type 2 inflammation induced by *Nippostrongylus brasiliensis*. We hypothesized that NAMs acquire epigenetically imprinted trained immunity and tested this in a heterologous challenge model in which mice infected with *Nippostrongylus brasiliensis* were challenged 4–6 weeks later with lethal H1N1 influenza. Remarkably, all *Nb*-conditioned mice survived, whereas all unconditioned controls succumbed by days 5–6 post-infection. Protection occurred without improved viral burden or enhanced T cell responses, and instead tracked with reduced immunopathology, amplified type 2 cues, increased efferocytosis and accelerated tissue repair. Using NAM-DTR mice, we show that conditioned NAMs are necessary and sufficient for protection: depletion or replacement with unconditioned NAMs abrogated survival, whereas adoptive transfer of conditioned NAMs conferred tolerance without enhancing viral clearance. Genomic analyses implicated an IL-4–STAT6–PPARγ and Arginase-1 chromatin program that imprints a pro-resolving and reparative NAM state driving programs of tissue repair, type 2 immunity and efferocytosis during lethal respiratory viral infections. Finally, meta-analysis of human lung single-cell atlases from healthy, IPF and COPD cohorts indicated that reparative NAM-like programs aligned with fibrotic remodeling in IPF but diverged in COPD, supporting context-dependent consequences of sustained repair states. These findings establish local trained immunity in lung-resident macrophages as a mechanism of disease tolerance and a therapeutic entry point for severe inflammatory respiratory infections.

An effective immune response requires balancing pro-inflammatory and regulatory signals, a task mediated by tissue-resident macrophages (RTMs) early during infections: which in lungs either refer to alveolar macrophages (AMs) or interstitial macrophages (IMs)^1–11^. IMs comprise functionally distinct subsets defined by markers such as CD169, CX3CR1, CD206, Lyve1, and MHCII^3,12,13^ and chemokine expression^14^. We identified a CD169^+^ IM subset positioned around large airways and pulmonary nerves in both mice and humans called nerve- and airway-associated interstitial macrophages (NAMs) that express wound-repair, immunoregulatory, and efferocytosis programs^3^. In contrast to AMs, which undergo substantial cell death after influenza or SARS-CoV-2 infection, NAMs expand early^3,15^, suggesting that they may undergo epigenetic changes to acquire hallmarks of trained immunity.

Trained immunity is a durable reprogramming of innate cells—through metabolic rewiring and epigenetic remodeling—following an initial stimulus^16–24^. Most work has focused on “central” training^20,25–28,22,29,30,31^, in which epigenetic changes in bone marrow precursors rewire monocytes, macrophages and neutrophils to enhance antimicrobial resistance, typically improving pathogen control^29,32–34^. By contrast, evidence for “local” trained immunity in resident macrophages that protects primarily through disease tolerance—limiting immunopathology rather than reducing pathogen burden—remains limited. Disease tolerance differs from resistance in that it preserves tissue function by restraining immune-mediated damage without necessarily lowering pathogen load.^31,35–41,42–44^. Dissecting disease tolerance is inherently challenging because it requires separating tissue-protective mechanisms from effects on pathogen clearance^41,45^.

In this study, we asked whether NAMs can acquire local trained immunity that promotes disease tolerance during lethal respiratory viral infection. We established a heterologous model in which mice were infected with *Nippostrongylus brasiliensis* (*Nb*), rested for four weeks, and then challenged with a lethal dose of influenza. We show that type 2 immune history can imprint a durable, locally trained state in nerve- and airway-associated macrophages (NAMs) that shifts the lung toward disease tolerance during subsequent severe respiratory viral challenge. This macrophage-centered program couples immunoregulation with inflammation resolution and tissue repair to limit immunopathology and preserve host function, establishing tissue-resident macrophage training as a mechanism by which prior immune experience shapes divergent infection outcomes. Together, our findings position NAMs as a key cellular node through which immune history calibrates mucosal resilience, and they highlight macrophage conditioning as a potential therapeutic strategy to promote tolerance in severe respiratory infections and other pulmonary inflammatory diseases.

## RESULTS:

### Prior *Nb* infection confers complete protection against lethal heterologous respiratory viral infection.

**Extended Data Fig.1:**
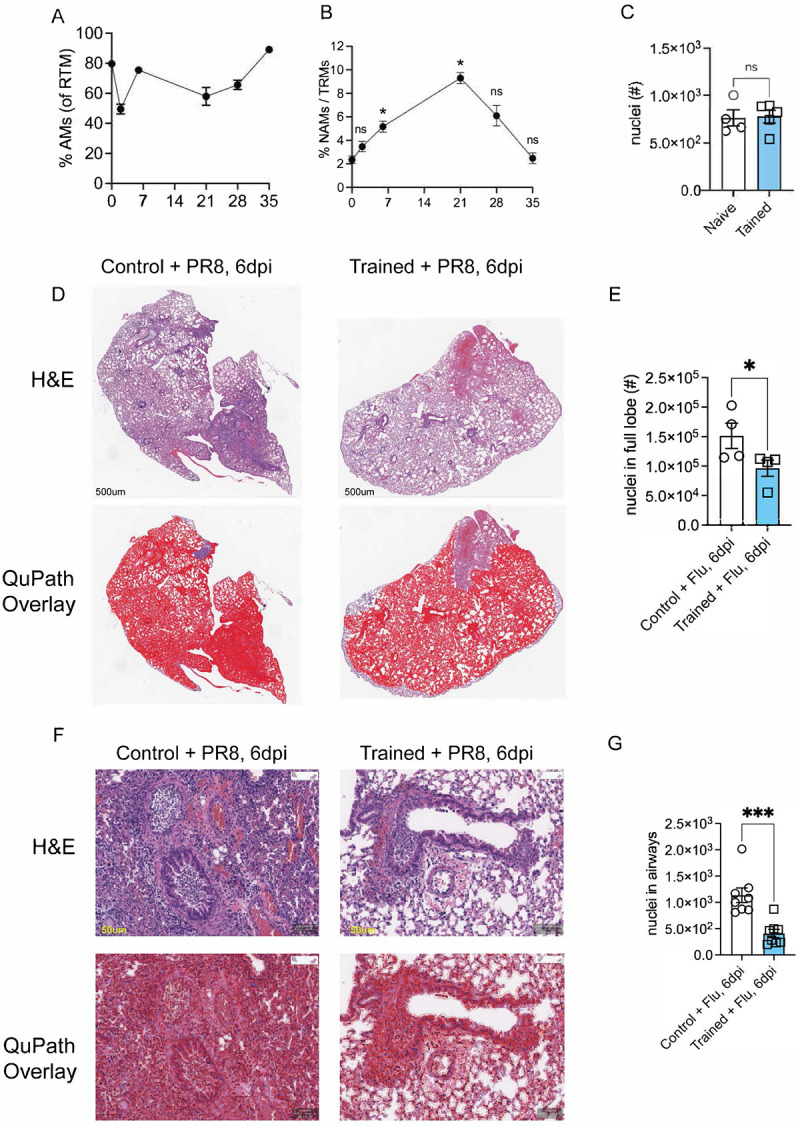
*Nb* exposure results in robust expansion of NAMs, but not AMs. Flow cytometric based kinetics analysis of lung lobes harvested from mice on days 0, 7, 14, 21, 28 and 35 post a single inoculation of 600-L3 stage *Nb* larvae injected subcutaneously under anesthesia. Mice were euthanized and lungs were harvested, digested with Liberase^™^ and single cell suspensions of flow cytometry were prepared as outlined in the Methods. Proportions of (A) AMs characterized by CD169^+^ Siglec-F+ CD11c+ CD11b+/− and (B) NAMs characterized by CD169^+^ Siglec-F^−^ CD11c^−^ CD11b^+^ CX3CR1^hi^ are represented as frequency of RTMs (MerTK+ CD64+ F4/80hi Ly6G- CD3- B220- NK1.1- Ly6C-). Total lung lobes were scanned and uploaded at high resolution (.svs) from Omero onto QuPath and total nuclei was calculated on analysis tool on QuPath for cell detection with detectable nuclei (C) QuPath analysis inflated lung sections from Fig.1F showing total nuclei in control (white bars) or trained (blue bars). Data represented as Mean±SEM, *n*=*5*. Statistics based on Student *t*-test (Mann-Whitney test). Total nuclei were calculated based on non-inflated H&E-stained lungs from control + PR8 and Trained + PR8 at 6dpi. (D) Image analysis scheme showing selected H&E stained lunts (top row) and cell density region of interest marked in red (bottom rows) for lung at 500 um. (E) Quantification of total nuclei based on QuPath analysis for infected control (white bars) and trained (blue bars). Data represented as Mean±SEM, *n*=*4*. Statistics based on student t-test (Mann-Whitney test). (F) QuPath ROI analysis as in (D) for airways mapped to quantify cellular infiltration. Multiple airways were tagged per each lung. (G) Quantification of total nuclei based on QuPath analysis for infected control (white bars) and trained (blue bars). Data represented as Mean±SEM, *n*=*7–8*. Statistics based on student t-test (Mann-Whitney test).

We began our study by determining how different pulmonary macrophage subsets respond to *Nb* infection. Using flow cytometry, we assessed the kinetics of AMs and NAMs for 4 weeks after an infection with L3-*Nb* larvae in wildtype (WT, C57Bl/6) mice. We found that frequency of AMs (percent of total RTMs: MerTK^+^ CD64^+^ F4/80^hi^) declined and rebounded by day 35 (Extended Data Fig. 1A). NAMs, in contrast, underwent a proliferative burst during the first three weeks and then returned to baseline by day 35 (Extended Data Fig. 1B) suggesting that *Nb* infection may “train” NAMs, priming them for a protective response in subsequent infections.

**Figure-1:**
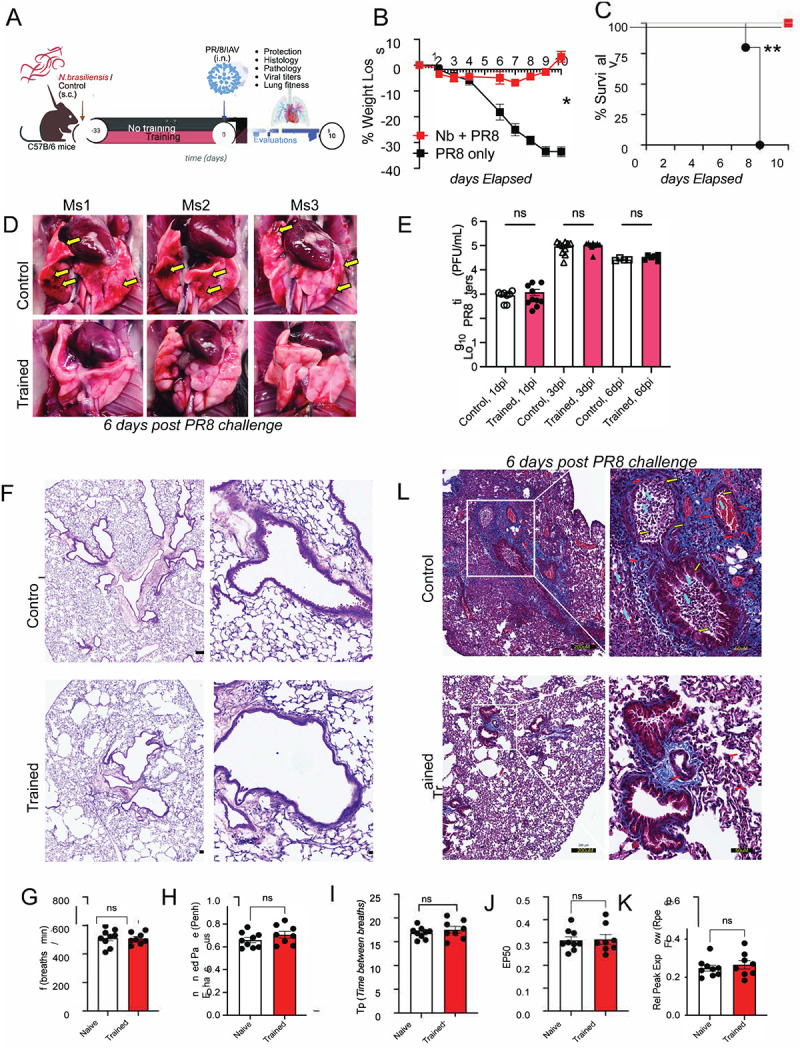
Prior *Nb* infection confers complete protection against lethal heterologous respiratory viral infection. (A) Model for heterologous infection of C57BL/6J mice with or without an inoculation of 600 L3-stage *Nb* parasites subcutaneously, followed by a 4–5-week resting period before an intranasal challenge of 150 EID50 PR8 strain H1N1 virus on day 33. Mice were monitored for 10 days following challenge and evaluated for (B) %Weight loss (Mann-Whitney test, *p<0.01) and (C) %Survival showing 100% mortality of control while 100% survival of *Nb-*trained mice after PR8 challenge (Kaplan-Meier test, *p<0.001). (D) Representative images of gross pathology of mouse lungs from infected mice at 6 days post PR8 challenge showing disease foci (yellow arrows), *n*=*5/group*. (E) Whole lung lysates were homogenized, and total infectious viral titers were quantified by plaque assay showing infectious PR8 in control or *Nb-*trained at 1-, 3- and 6-days post challenge (Mann-Whitney test, *n*=*5–10/group*). (F) Lungs were expanded at 10psi and fixed, paraffin embedded, sectioned at 5um and stained for Hematoxylin and Eosin (H&E) for control (top row) and *Nb-*trained (bottom row) at a scale of 200μm (left images) and close-up 50μm insets (right images) showing that control and *Nb-*trained lungs look similar in morphology prior to PR8 challenge. (G-K) Whole body plethysmography analysis of naïve and *Nb-*trained mice (as in A) showing no differences in key parameters regarding lung fitness and function such as (G) *f,* breaths/min, (H) *PenH,* Enhanced Pause, (I) *Tp,* times between breaths, (J) *EP50,* time required to exhale 50% of the breath and (K) *Rpef,* Relative Peak Expiration Flow (n=5/group). (H) Masson’s Trichrome stained 5um lung sections of control and *Nb-*trained (bottom) at a scale of 200μm (left images) and expanded 50μm insets (right images). Data are representative of at least 2 experiments (n= 4–10/group). Values represent the mean ± SEM. Schematic in (A) created using Biorender.com.

To test this, we utilized a heterologous infection model in which mice were infected with ~600 L3-stage *Nb* larvae. Four weeks later, these mice were challenged with a lethal dose of the H1N1 influenza virus, specifically the A/Puerto Rico/8/34 strain (hereafter referred to as PR8) (Fig. 1A). Mice previously infected with *Nb* are referred to as “trained mice,” while those not infected are designated as “control mice”. We observed that control mice lost over 20% of total body weight within the first week following PR8 challenge. In contrast, trained mice experienced significantly less weight loss and rapidly returned to their baseline weight by 8 days post viral challenge (Fig. 1B), protecting them from mortality. Notably, all control untrained mice reached the clinical endpoint (Fig. 1C). Gross lung pathology at 6 days post infection (dpi) revealed stark differences between trained and control mice. The lungs of trained mice appeared healthy and showed signs of full recovery, whereas the lungs of untrained mice exhibited severe acute lung injury, including pneumonia, vascular damage, and hemorrhaging (Fig. 1D). To determine whether protection reflected enhanced pathogen control, we quantified infectious virus and observed no differences between trained and control mice at 1, 3, or 6 dpi (Fig. 1E). At baseline, naïve and trained lungs were indistinguishable prior to PR8 challenge with emergent emphysema in trained mice which is a consequence of *Nb* infection (Fig. 1F) with no infiltrating immune cells in the lung parenchyma by measure of total nuclei detected using QuPath^™^ analysis (Extended Data Fig. 1C). Moreover, we performed whole-body plethysmography (WBP) on naïve and *Nb*-trained mice at day 35 to ensure that *Nb* training did not alter baseline pulmonary function. Because lung fitness and gas-exchange capacity are multifactorial—captured by 11 parameters in prior studies^46–50^ —we focused on respiratory rate (f; Fig. 1G), enhanced pause (*PenH*; Fig. 1H), expiratory pause (*Tp*; Fig. 1I), the volume at 50% expiratory flow (*EF50*; Fig. 1J), and peak expiratory flow rate (*Rpef;* Fig. 1K). None of these measures differed significantly between naïve and trained mice, indicating comparable baseline lung function. We next examined paraffin-embedded lung sections collected at 6 days post viral infection (dpi) by H&E staining (Extended Data Fig. 1C) and Masson’s trichrome staining (Fig. 1L).

After PR8 challenge, lung pathology diverged sharply between untrained and trained mice in both peribronchial (PB) and alveolar compartments. Peribronchially, control mice displayed (1) darker thicker bands of blue collagen staining (red arrows), consistent with collagen cuffing/expanded PB regions linked to fibrosis, reduced elasticity, and impaired bronchial function, whereas trained mice showed lighter, thinner collagen deposits. We found^51^ disrupted mesenchymal organization (yellow arrows) in control mice, while trained mice preserved the characteristic columnar/cylindrical epithelial morphology. We found (3) a prominent infiltration of red blood cells and inflammatory cells in the PB space (Cyan arrows). In the alveolar compartment, control mice exhibited increased RBC leakage and inflammatory infiltration consistence with diffuse alveolar hemorrhaging, which was markedly reduced in trained mice, indicating reduced immunopathology and tissue preservation (Fig. 1G). We quantified the H&E sections in two ways. First, we mapped the lung lobe as a single region of 500um^52^ and then using QuPath^™^ calculated total nuclei in control (left) and trained (right) at 6dpi (Extended Data Fig. 1D) and quantified total nuclei (Extended Data Fig. 1E) showing significantly more infiltrating cells in control compared to trained 6d post flu challenge. Second, we focused on airways around 50um regions and calculated nuclei within each airway for the two cohorts for control (left) and trained (right) 6dpi (Extended Data Fig. 1F). Our automated software analysis on nuclei within the airways showed significantly more infiltration in control compared to trained cohorts (Extended Data Fig. 1G). Together, these data show that prior Nb infection confers robust protection from lethal PR8 challenge by preserving lung architecture and limiting pathological injury without altering viral loads.

### Enhanced type 2 immune response in *Nb* trained mice following PR8 viral challenge

**Figure-2:**
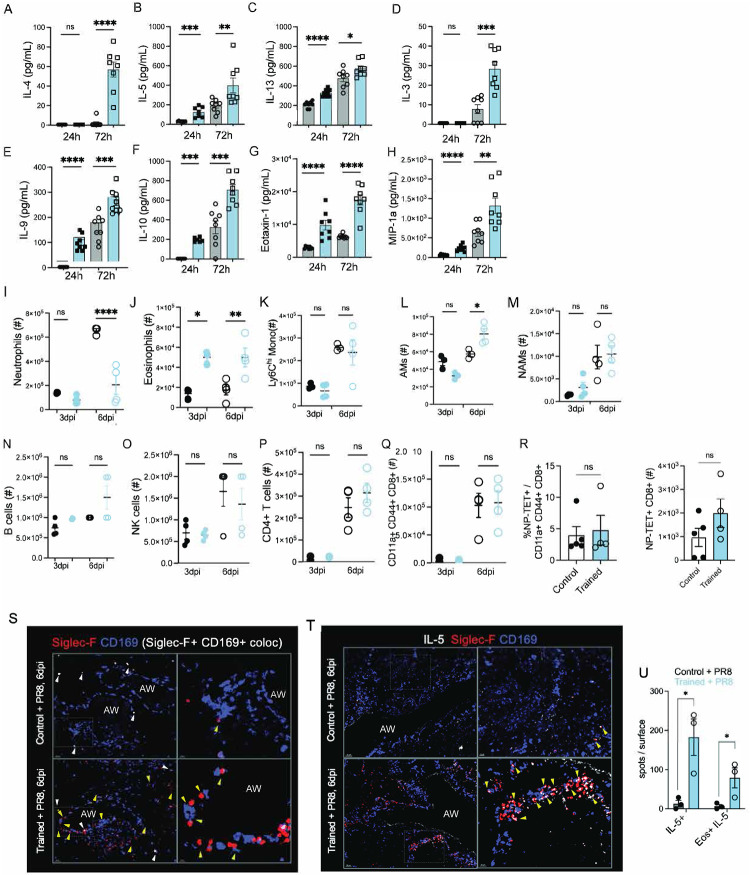
Enhanced type 2 immune response in *Nb* trained mice following PR8 viral challenge. (A-H) Cytokine analyses of lung lysates in Control (grey bars, circles) and *Nb-*trained (blue bars, squares) mice at 24h or 72h post influenza challenge with PR8. (A-G) Type 2 cytokines shown for quantification of IL-4, 5, 13, 3, 9, 10 and Eotaxin-1. (H) Quantification of macrophage associated cytokine MIP-1a. (I-R) Flow cytometric analyses of whole lungs from WT (black circles) or *Nb-*trained (red circles) mice on 3 days challenge (solid circles) or 6 days (open circles) post 150 EID50 PR8-IAV infection. Total counts shown for (I) Neutrophils (Ly6G+ CD11b+), (J) Eosinophils (Siglec-F+ CD11c- CD11b+ Ly6G-), (K) Ly6Chi Monocytes (Ly6C++ MHCII- CD11b+), (L) AMs (CD169+ Siglec-F+ CD11c+ CD11b+/− Ly6C-), (M) NAMs (CD169+ Siglec-F- CD11c- CD11b+ CX3CR1+ Ly6C-), (N) B cells (CD19+), (O) NK cells (NK1.1+ CD3-), (P) CD4+ T cells, (Q) Activated CD8+ T cells (CD8+ CD11a+ CD44+, CD62L-) and (R) NP-Tetramer+ CD8+ T cells. Data are representative of two independent experiments and shown as mean ± SEM (n=3–5/group; ns = not significant, *p< 0.01, **p<0.001, ****p<0.0001, One way ANOVA – Kruskal-Wallis Test). Representative confocal images of 20-micron lung sections of PR8 infected Control^52^ or *Nb-*trained (bottom) mice at 6dpi showing (S) AMs (white cells and arrows, CD169+ Siglec-F+ coloc), NAMs (blue cells decorating large airways (AW) and eosinophils (red cells, yellow arrows; CD169- Siglec-F+). Scale is 30um (left) and 10um (right). and (T) Representative confocal images of 20-micron lung sections of PR8 infected Control^52^ or *Nb-*trained (bottom) mice at 6dpi showing IL-5 (white cells), NAMs (blue cells decorating large airways (AW) and eosinophils (red cells, yellow arrows; CD169- Siglec-F+). Scale is 30um (left) and 10um (right). Yellow arrows point to eosinophils co-localized with IL-5. Data representative of 2 independent experiments, each with *n*=*3*. (U) IMARIS^®^ (Version 8.3.1) based quantification of total Eos (red cells) and IL-5 (white cells) as in (T) as calculated from surfaces of proximity to large airways from 3 lungs. Data presented as Mean±SEM and statistics determined by Student *t-*test (Mann-Whittney test), *p=*0.03*.

To investigate the mechanisms of how *Nb* training enhances protection against respiratory viral infection, we first measured the lung cytokines production early after PR8 challenge. Because type 2 cytokines (e.g., IL-4, IL-13) promote tissue repair^53–59^ and suppress excessive neutrophil activity^51,60–64^, we hypothesized that *Nb* training may lead to enhanced production of type 2 cytokines early after PR8 challenge. To test this, we harvested and homogenized whole lungs from trained and control mice at 24 and 72h post PR8 challenge and analyzed their cytokine and chemokine production using 23-plex Luminex assay. Compared to control mice, trained mice showed a remarkably higher production of type 2 cytokines such as IL-4, 5, 13, 3 and 9 as early as 3 days after PR8 challenge (Fig. 2A–E). Furthermore, we found significantly higher secretion of anti-inflammatory cytokine IL-10 in trained lungs compared to control mice (Fig. 2F). Intriguingly, we observed significantly higher levels of Eotaxin-1 production (Fig. 2G) in trained mice following PR8 challenge. An increase in both IL-5 and Eotaxin-1 may explicate the enhanced eosinophil recruitment in the lungs of trained mice. We also found an increase in the production of Macrophage Inflammatory Protein 1 alpha (MIP-1a) (Fig. 2H), which is associated with recruitment of macrophages to the site of PR8 infection. However, we found no significant differences in secretion of inflammatory cytokines such as in IL-1β (Extended Data Fig. 3A) or IFN-α (Extended Data Fig. 3B).

**Extended Data Fig.2:**
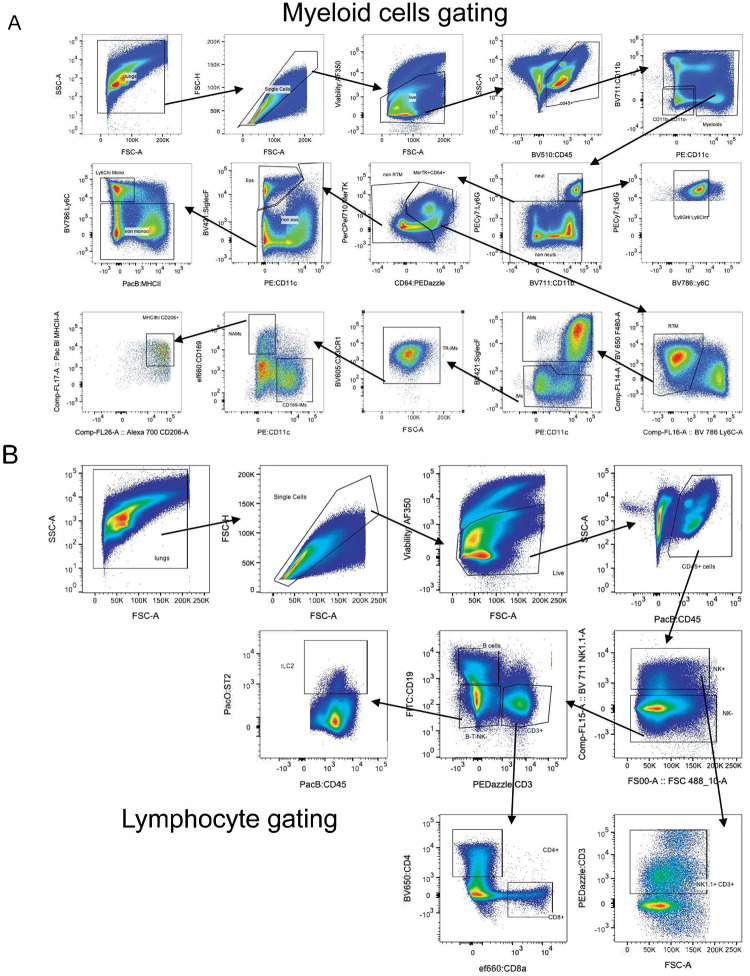
Flow cytometry gating strategy to represent lung immune cells. (A) Representative flow gating strategy shown for myeloid panel with identified populations of total myeloid cells, neutrophils, resident tissue macrophages, eosinophils, Ly6Chi monocytes, AMs, tissue resident interstitial macrophages (TR-IMs) and NAMs. (B) Representative flow gating strategy shown for B cells, CD4+ T cells, CD8+ T cells and NK cells.

**Extended Data Fig.3:**
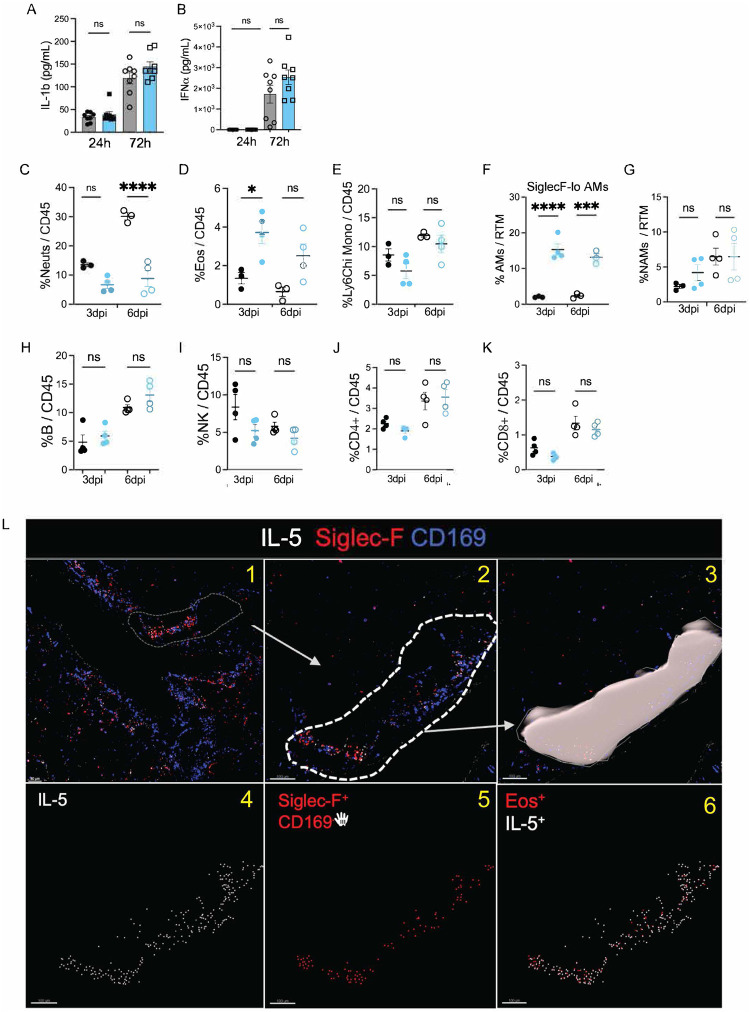
Innate and adaptive cellular regulation is mediated by enhancement of eosinophilia and NAMs following PR8 infection. (A-B) Cytokine analyses of lung lysates in Control (grey bars, circles) and *Nb-*trained (blue bars, squares) mice at 24h or 72h post PR8 challenge with PR8. (C-K) Flow cytometric analyses of whole lungs from WT (black circles) or *Nb-*trained (blue circles) mice on 3 days challenge (solid circles) or 6 days (open circles) post 100 EID50 PR8-IAV infection. Frequency of CD45% cells for (C) Neutrophils (Ly6G+ CD11b+), (D) eosinophils (Siglec-F+ CD11c- CD11b+ Ly6G-), (E) Ly6Chi Monocytes (Ly6C++ MHCII- CD11b+), Frequency of RTM for (F) AMs (CD169+ Siglec-Flo CD11c+ CD11b+/− Ly6C-), (G) NAMs (CD169+ Siglec-F- CD11c- CD11b+ CX3CR1+ Ly6C-), Frequency of CD45% (H) B cells (CD19+), (I) NK cells (NK1.1+ CD3-), (J) CD4+ T cells and (K) CD8+ T cells. Data are representative of two independent experiments and shown as mean ± SEM (n=3–5/group; ns = not significant, *p< 0.01, **p<0.001, ****p<0.0001, One way ANOVA – Kruskal-Wallis Test). (L) Confocal imaging showing IMARIS^®^ quantification scheme for total IL-5 and Siglec-F+ CD169- IL-5+ cells. Steps in order (1–6) explaining how (1) Regions of interest was selected,^51^ labeled (3) converted into a surface (4) each IL-5+ signal was assigned as a white spot and (5) each eosinophil (Siglec-F+ CD169-) was assigned a red spot and (6) white and red spots were co-analyzed for Eos+ IL-5+ and were subsequently calculated.

We profiled innate and adaptive lung immune responses in control and *Nb*-trained mice at 3 and 6 days after PR8 infection by multiparametric flow cytometry (Extended Data Fig. 2A, B). Flow analysis of Liberase^™^-digested lungs^3,15^ revealed a significantly higher accumulation of neutrophils (Ly6G+ CD11b+) in control mice compared to trained mice at 6dpi (Fig. 2I, Extended Data Fig. 3C). In contrast, eosinophils (Ly6G^−^ CD11b^+^ Siglec-F^+^ CD11c^−^) were increased in trained mice (Fig. 2J, Extended Data Fig. 3D) at both 3 and 6 days post viral challenge. We observed no significant differences in numbers or frequency of infiltrating Ly6C^hi^ Monocytes (Fig. 2K, Extended Data Fig. 3E). In comparison By comparison, trained mice showed increased numbers of resident Siglec-F^hi^ CD11c^+^ CD11b^−^ AMs (Fig. 2L) as well as bone marrow (BM)-derived Siglec-F^lo^ CD11c^+^ CD11b^+^ AMs (Extended Data Fig. 3F) which suggests reduced AM loss^65–68^ after PR8 challenge in trained lungs. Finally, consistent with prior work showing robust NAM proliferation after flu infection^3^, NAMs increased in numbers in both control and trained mice following PR8 challenge (Fig. 2M, Extended Data Fig. 3G), with a trend toward higher counts in *Nb*-trained mice that did not reach statistical significance.

**Figure-3:**
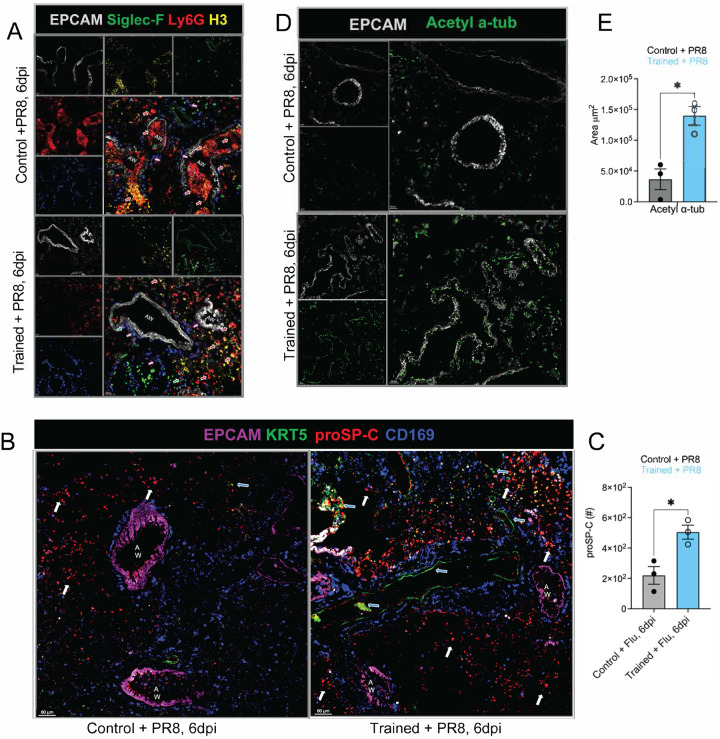
*Nb* training results in a distinct spatial organization of innate immune cells in virally infected lungs resulting in superior tissue repair and resolution. Representative confocal images of 20-micron lung sections of PR8 infected control (top panels) or *Nb-*trained (bottom panels) mice at 6dpi. (A) Evaluation of extent and location of NETs formation using EPCAM (light grey) to mark the epithelium and large airways (AW), NAMs (Siglec-F- CD169+, blue), Neutrophils (Ly6G, red) and NETs (in green). Scale bar represents 100 μm. Red arrows point to the neutrophil and NET filled airways; White arrows point to NAMs. (B) Evaluation of tissue repair and resolution using NAMs (blue, CD169+) in dendritic morphology, KRT5 (green, blue arrows) and proSP-C (red, white arrows). Scale bar represents 80 μm. Representative images from 2 individual experiments each with n=2–3. (C) Corresponding quantification of proSP-C from control and train cohorts done using IMARIS software. (D) Evaluation of epithelium and preserved cilia using EpCam (in light grey), Acetylated α-tubulin (in green) and NAMs (blue, CD169+) in dendritic morphology for 20um lung sections from naïve (top row), or infected control at 6dpi (middle row) or infected trained mice at 6dpi (bottom row). Scale bar represents 50 μm. (E) Corresponding quantification of Acetylated α-tubulin done using IMARIS software. Representative images from 2 individual experiments each with n=3. Corresponding quantification of Acetylated α-tubulin^160^ from both cohorts. Data presented as Mean±SEM, n=3/group. Statistical analysis determined using Student *t-*test (Mann-Whitney test), *p=*0.05*.

We found no differences in number or frequency of B cells, NK cells, CD4^+^ T cells (Fig. 2N–P, Extended Data Fig. 3H–J) in either cohort. We also did not find any statistical differences in the frequency of CD8^+^ cells (Extended Fig. 3K) or the frequency of activated CD8^+^ T cells (CD11a^+^ CD44^+^ CD62L^−^) (Fig. 2Q) or NP-Tetramer^+^ CD8^+^ T cells (Fig. 3R). These data argue against a major contribution of adaptive immunity to the protection observed in *Nb*-trained mice following PR8 challenge. Collectively, our results indicate that *Nb* conditioning sustains an early type 2 immune program after PR8 challenge, dampens neutrophil accumulation, and preserves lung architecture through predominantly innate mechanisms.

### *Nb* training results in a distinct spatial organization of innate immune cells in virally infected lungs resulting in superior tissue repair and resolution.

We next asked whether the reciprocal neutrophil–eosinophil changes detected by flow cytometry (Fig. 2I–J, Extended Data Fig. 3C–D) were reflected in altered spatial organization within infected lungs of control and trained mice. Using multiparametric confocal imaging of lung sections at 6 dpi, we resolved three myeloid populations: NAMs (Siglec-F^−^ CD169^+^; blue), alveolar macrophages (AMs; Siglec-F^+^ CD169^+^; white, arrows), and eosinophils (Siglec-F^+^ CD169^−^; red, yellow arrows). NAM abundance was comparable between cohorts after infection, whereas eosinophils were markedly increased in trained lungs and clustered near NAMs—a pattern not observed in controls. Notably, majority of the eosinophils (red cells) were observed interacting closely with NAMs (blue cells) along the large airways shown in yellow arrows found in trained mice but not in control mice (Fig. 2S, insets on right). Given the elevated IL-5 production in trained mice (Fig. 2B), we next stained for IL-5 and found robust IL-5 signal along large airways in trained lungs, where it colocalized primarily with eosinophils (Siglec-F^+^ CD169^−^), but was largely absent in control mice (Fig. 2T). We quantified total IL-5 and the fraction of IL-5 signal colocalized within eosinophils by generating surface masks along the large airways. We then assigned each IL-5 signal (white spot) and each eosinophil (Siglec-F^+^ CD169^−^; red spot) and quantified the number of colocalized eosinophil:IL-5 events within the masked surface (Extended Data Fig. 3L). This analysis revealed significantly higher total IL-5 protein levels, along with significantly increased colocalization of IL-5 with eosinophils (Siglec-F^+^ CD169^−^) (Fig. 2U). As noted in Fig.2S majority of the IL5^+^ eosinophils were again observed interacting closely with NAMs (blue cells) along the large airways (Fig. 2T **lower panels**). These data show that eosinophils are the predominant source of IL-5 in the trained lungs —a response that is absent in control mice following PR8 infection. Together, these data indicate that *Nb* training establishes a type 2–skewed spatial niche after PR8 challenge, characterized by eosinophil enrichment and the airway-associated IL-5 production underpinning enhanced disease tolerance.

**Extended Data Fig.4:**
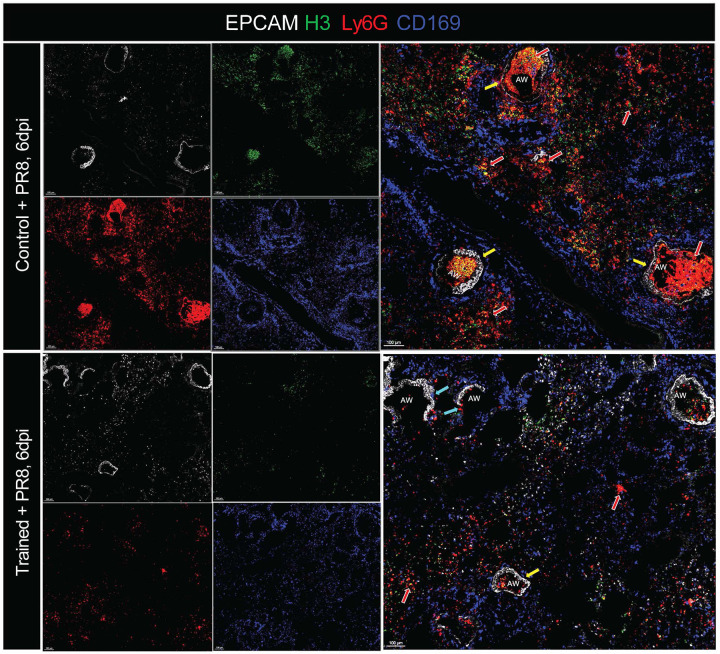
Control lungs show exacerbated NETosis in airways following PR8 challenge, which is absent in *Nb-*trained lungs. (A) Representative confocal images of 20-micron lung sections of PR8 infected control^52^ or *Nb-*trained (bottom) mice at 6d post PR8 challenge. Evaluation of extent and location of NETs formation was performed using EPCAM (light grey) to mark the epithelium and large airways (AW), Ly6G (red), H3 (yellow), H3^160^ and CD169 (blue). Red arrows point to the neutrophil and NETs clusters, yellow arrows point to the epithelium. Representative of 2 individual experiments each with n=3.

In parallel, we observed pronounced neutrophil accumulation in control mice after PR8 infection, consistent with links between excessive neutrophilia and poor infection outcomes^69–74^. Immunostaining of lung cryosections at 6 dpi showed dense Ly6G^+^ neutrophil aggregates within airways of control mice, whereas trained lungs contained sparse neutrophils (Fig. 3A, top and bottom), consistent with our flow cytometric data (Fig. 2I). To assess neutrophil extracellular traps (NETs), we stained for histone H3 (green/yellow) together with CD169 (blue) to mark resident tissue macrophages and EPCAM (light grey) to outline airways. Control lungs exhibited abundant airway and alveolar neutrophils with prominent NET-associated signal (Fig. 3A, **yellow and red arrows**), features that were largely absent in trained lungs. Importantly, these differences occurred without detectable changes in the NAM proliferative burst after infection (Extended Data Fig. 4).

Given the striking tissue injury in controls but not trained mice at 6 dpi (Fig. 1F, G and Extended Data Fig. 1C) we next assessed early regenerative programs. Lung tissue repair depends on the expansion of regenerative cytokeratin-5+ (KRT5) cells^75–77^, cytokeratin 8 (KRT8)^78^ as well as secretion of surfactants such as pro-surfactant protein C (proSP-C)^79,80^ that precede the expansion of KRT5+ cells. To determine whether *Nb*-training enhanced the lung’s regenerative potential, we stained lung sections for KRT5 and proSP-C. Staining for proSP-C and KRT5 revealed emerging KRT5^+^ cells in trained lungs that overlapped with proSP-C (Fig. 3B, **blue arrows**), whereas control lungs showed markedly fewer proSP-C^+^ cells shown by white arrows (Fig. 3B). Imaris based quantification of proSP-C showed a significantly higher number of proSP-C cells in trained mice compared to controls after PR8 infection (Fig. 3C), consistent with enhanced early regenerative potential in trained mice.

Finally, to evaluate airway epithelial differentiation and repair, we stained for EPCAM and acetylated α-tubulin (Ac α-tub), a marker of multi-ciliated airway epithelial cells that increases during post-PR8 epithelial restoration^81^. Indeed, following infection, Ac α-tub signal was significantly stronger and more widespread in trained lungs when compared to control lungs across both small and large airways, often in regions proximal to NAMs (Fig. 3D, **top vs. bottom panels**). Imaris based quantification of Ac α-tub showed a 3-fold increase in trained mice compared to controls (Fig. 3E). These findings are consistent with increased ciliated cells and enhanced repair of conducting airways at 6 days post-PR8 challenge. Collectively, these imaging data show that *Nb* training reshapes innate immune cell positioning—enhancing eosinophil/IL-5 airway niches while limiting neutrophil and NET accumulation—and is associated with early activation of regenerative epithelial programs that align with improved tissue preservation and recovery.

### Locally trained NAMs are necessary and sufficient for promoting disease tolerance and ensuring host survival during a lethal influenza viral challenge.

**Extended Data Fig. 5:**
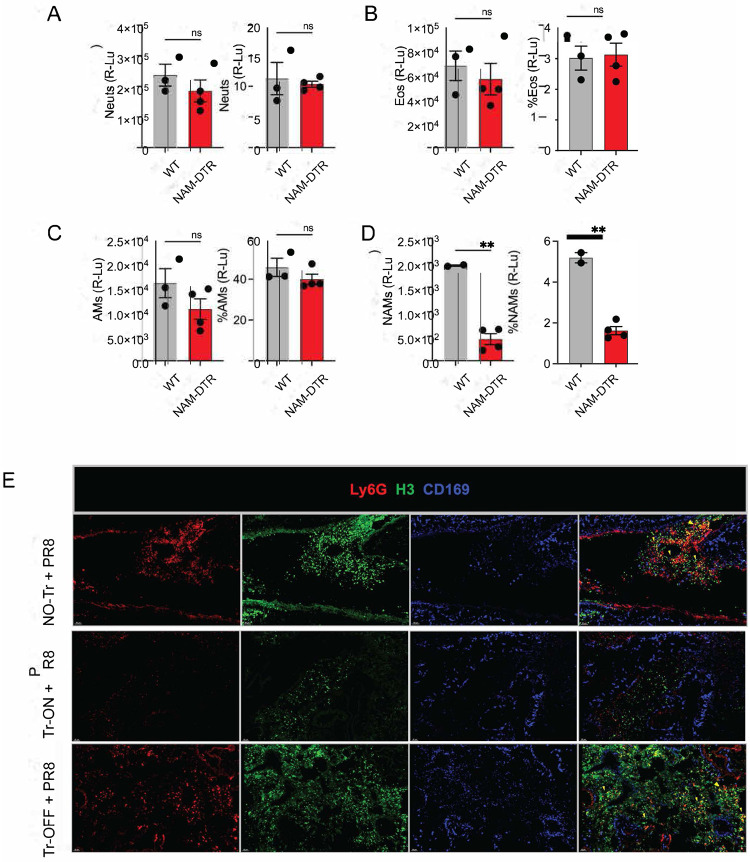
Validation of NAM-DTR transgenic mice system and resulting lung inflammation during flu challenge. WT or NAM-DTR mice were injected with DT intraperitonially and harvested 48h later to evaluate lung immune profile for total counts or frequency from CD45+ cells for (A) neutrophils, (B) eosinophils or frequency of total RTMs (MerTK+ CD64+ F4/80Hi Ly6Cneg) for (C) AMs and (D) NAMs from control (grey bars) or NAM-DTR mice (red bars). Data shown as mean ± SEM (n=3–4/group; ns = not significant, **p<0.001. One way ANOVA – Kruskal-Wallis Test). (E) Representative confocal images of 20 micron lung sections stained for neutrophils (Ly6G, red), NETs (H3, green) or NAMs (CD169, blue) for untrained controls (NO-Tr, top row), *Nb-*trained WT treated with DT (Tr-ON, middle row) and *Nb-trained* NAM-DTR mice treated with DT (Tr-OFF showing NAM-OFF, bottom row) – then all mice infected with PR8 on day 33 and evaluated 3 days later. Left three panels show individual stains of Ly6G, H3 and CD169 and panel on the right shows a merge highlighting NETs and Neutrophils that overwhelm lung parenchyma (yellow arrows showing H3+Ly6G+ yellow clusters) in NO-Tr and Tr-OFF cohorts but not Tr-ON mice (n=3/group).

Our findings raised a central question: which cells mediate the protection observed after heterologous viral challenge in *Nb*-trained mice? We therefore sought to identify the macrophage subset(s)—or other immune populations—responsible for this effect. Protection conferred by *Nb* conditioning was not explained by enhanced viral clearance but instead reflected improved immunoregulation during lethal respiratory viral infection. Given that NAMs expand early after *Nb* infection and exhibit immunoregulatory and tissue-repair programs consistent with innate immune training, we leveraged our NAM-DTR model l (*CD169-cre* mice to *B6N.129P2-Cx3cr1*^*tm3 (DTR)Litt*^*/J* mice)^3,15^ to selectively deplete only the trained NAMs while sparing AMs or other immune or non-immune cells. To validate specificity and efficiency, we administered diphtheria toxin to WT and NAM-DTR mice and quantified lung immune populations two days later; NAMs were selectively ablated in NAM-DTR mice, with neutrophils, eosinophils, and AMs unaffected (Extended Data Fig. 5A–D).

**Figure-4:**
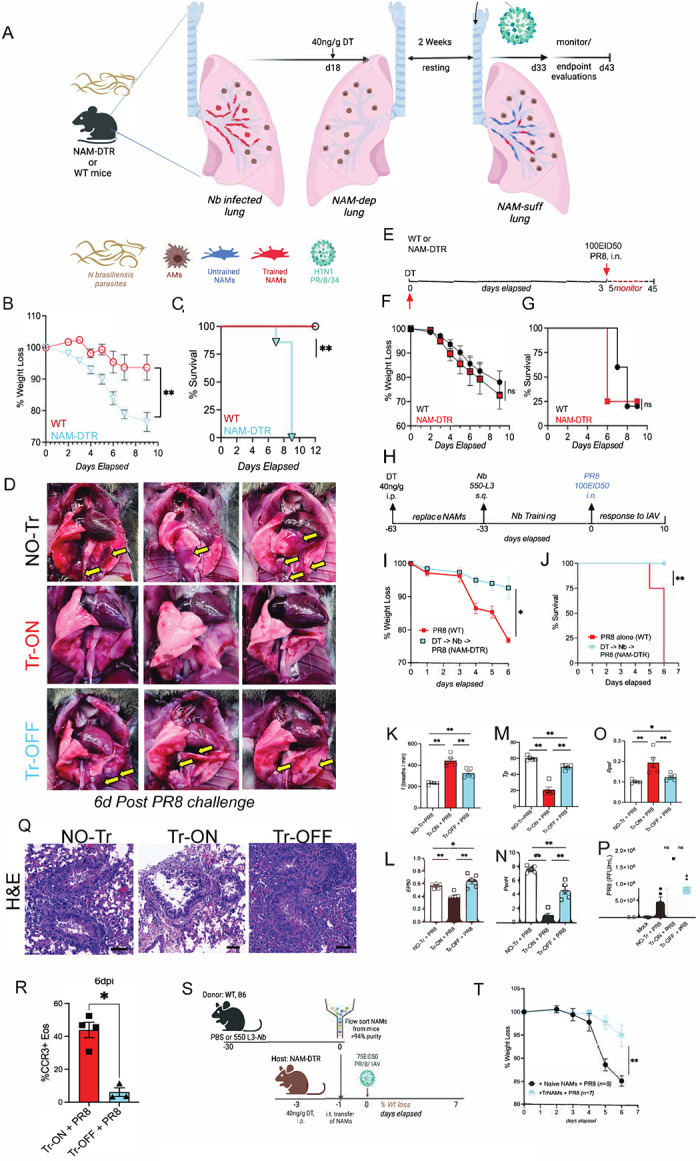
Depletion of *Nb-*trained NAMs abrogates anti-flu protection causing increased pathology, mortality correlating with increase inflammation. WT and NAM-DTR mice were inoculated with a 550–600-L3 stage *Nb-*larvae subcutaneously. On day 18, 40ng/g of DT was administered (i.p.) to all mice, then mice were let to rest until day 33. All mice were then challenged with 100EID50 of PR8 (i.n.) and cohorts of mice were monitored for additional 10 days. (A) Experimental scheme (B) %Weight loss in WT (red) or NAM-DTR mice (cyan) following PR8 challenge, shown as percentage of average weight. *n*=5/group; 2 independent experiments. Statistical significance was determined by paired *t-*test (Wilcox test) and Mean±SEM. **p=*0.0078*. (C) Kaplan-Meier of %Survival curves for WT and NAM-DTR mice following PR8 challenge. *n*=5/group; 2 independent experiments. Statistical significance was determined by Mantel-Cox test. **p=*0.0051*. (D) Gross pathology shown for *Nb-*trained WT mice (Tr-ON, top row), untrained WT mice (NO-Tr, middle row) and *Nb-*training OFF on NAM-DTR mice (Tr-OFF, bottom row) mice at 6 days post PR8 infection. Arrows depict lung damage and visible pneumonia forming. WT and NAM-DTR mice were treated with a single shot of 40ng/g DT intraperitonially (day 0) and mice were let to rest for 35 days. Then, all mice were infected with 100 EID50 PR8 intranasally. WT or NAM-DTR mice were treated with DT (40ng/g) and let to rest for 35 days, then infected with a lethal dose of PR8 intranasally and monitored for 10 days. (E) Experimental scheme (F) %Weight loss following PR8 challenge, shown as percentage of average weight. Statistical significance was determined by paired *t-*test. Mean ± SEM. (G) Kaplan -Meier of %Survival curves of WT and NAM-DTR mice following PR8 challenge. *n*=4/group. Statistical significance was determined by Mantel-Cox test. WT and NAM-DTR mice were inoculated with a 550-L3 stage *Nb-*larvae subcutaneously. WT or NAM-DTR mice were treated with DT (40ng/g) and let to rest for 30 days, then infected with 550 L3-*Nb* parasites (s.c.) and let to rest for additional 33d. Then, all mice were infected with 100EID50 PR8 (i.n.) and monitored for 6 days. (H) Experimental scheme (I) %Weight loss in WT (trained, blue lines) and NAM-DTR (NAMs depleted and replaced prior to *Nb* training, red lines) showing following PR8 challenge, shown as percentage of average weight. *N*=*4–5* /group; Statistical significance wsa determined by paired *t-*test (Wilcox test) and Mean±SEM. *p=*0.05*. (J) Kaplan-Meier of %Survival curves for WT and NAM-DTR (as in I). n=4–5/group. (K-O) Whole body plethysmography as described in Fig. 1 indicating lung-fitness of the 3 mouse cohorts as in (A) on 6d post PR8 challenge in the context of (K) *f,* (L) *EP50*, (M) *Tp*, (N) *PenH* and (O) *Rpef*. *p<0.01, **p<0.001, ***p<0.0005. (P) Lung lysate viral titers by plaque assay showing infectious PR8 in Mock-uninfected (open circles), infected mice from cohorts of NO-Tr (grey bars, filled circles), Tr-ON (red bars, filled black squares) and Tr-OFF (blue bars, filled triangles) at 3 days post PR8 challenge. Data represented as Mean+SEM (*n*=5/group). Statistical significance was determined by one-way Anova test (Kruskal-Wallis test). (Q) Representative H&E-stained lung sections shown from naïve, and PR8 infected control (NO-Tr), *Nb-*trained (Tr-ON) and *Nb-*trained NAMs replaced with untrained NAMs (Tr-OFF) (n=3). (R) Flow cytometry analysis of the two cohorts (as in B) on day 6 post PR8 challenge showing frequency of CCR3^+^ Eos (of total eosinophils, Siglec-F+ CD11b+) of Tr-ON (red bars with filled black squares) or Tr-OFF (blue bars with filled black triangles) on 3d post PR8 challenge. (S,T) WT mice were treated with PBS or infected with 550–600 L3-*Nb* parasites (s.c.) and mice were let to rest for 30 days. On day 30, naïve (PBS-treated) or trained (*Nb-*infected) lungs were harvested and single cell suspensions were collected, washed, counted, stained and flow purified to collect >95% pure NAMs. Donor NAM-DTR mice were treated with 40ng/g DT 48h prior and 10–20K NAMs were counted and intratracheally administered into anesthetized mice and all mice were subsequently infected with 75EID50 PR8 virus intranasally 14h later. (S) Experimental Scheme (T) %Weight loss for NAM-niche depleted NAM-DTR mice receiving naïve NAMs (black lines filled black circles) or trained NAMs (cyan line with cyan half-filled squares). Statistical significance was determined by paired *t-*test (Wilcox test) and Mean±SEM. *p=*0.01 (n*=*3–4/mouse)*.

We infected WT and NAM-DTR mice with *Nb* (**scheme shown in** Fig. 4A) and allowed 18 days for parasite clearance and restoration of steady-state lung conditions. Mice then received diphtheria toxin (DT; 40 ng/g, intraperitoneally), which selectively depleted *Nb*-conditioned NAMs in NAM-DTR mice while leaving NAMs in DT-treated WT controls intact. We then waited an additional two weeks to permit repopulation of the NAM niche from local and/or bone marrow–derived precursors, generating a replenished NAM compartment that had not experienced *Nb* conditioning (“untrained”). Mice were subsequently challenged with a lethal dose of PR8 (Fig. 4A) and monitored for several days. We reasoned that if protection is mediated primarily by local training of NAMs—rather than central training of bone marrow precursors^21,82^ —then selective removal of conditioned NAMs would eliminate protection. Consistent with this, *Nb*-trained WT mice (“Training-ON,” Tr-ON) treated with DT showed only modest transient weight loss, whereas DT-treated *Nb*-conditioned NAM-DTR mice (“Training-OFF,” Tr-OFF) exhibited pronounced weight loss (Fig. 4B), severe clinical disease, and reached humane endpoints by day 8 post-infection (Fig. 4C). Gross inspection of lungs at 6 dpi revealed severe pneumonia and hemorrhage in untrained controls (No-Tr) and Tr-OFF mice but comparatively preserved lung appearance in Tr-ON mice (Fig. 4D). Together, these data show that selective depletion of conditioned NAMs—followed by replacement with unconditioned NAMs—abrogates the protective effect of *Nb* conditioning despite intact AMs and other immune/stromal compartments. Thus, NAMs are the dominant macrophage population mediating *Nb*-induced disease tolerance during lethal influenza viral challenge.

To exclude the possibility that the phenotype in Fig. 4A reflected intrinsic properties of bone marrow–derived monocyte/macrophage precursors that repopulated the NAM niche after DT-mediated depletion, we depleted NAMs in naïve NAM-DTR mice and then rested the animals for 30 days to allow complete repopulation of the NAM compartment from bone marrow–derived precursors (Fig. 4E). We subsequently challenged these NAM-replenished mice with PR8 influenza. Interestingly, there were no differences in disease outcomes, including weight loss (Fig. 4F) or survival (Fig. 4G) between mice with monocyte-derived NAMs and those with resident NAMs. These findings demonstrate that *Nb*-induced training of NAMs represents a case of local innate immune training, rather than central immune training. Furthermore, they reveal that bone marrow derived replenished NAMs have similar functional capacity as the embryonic derived resident NAMs in response to influenza viral infection.

To exclude the possibility that NAM-DTR mice did not possess inherent defects in their ability to be *Nb* trained as with WT mice, we DT treated naïve and NAM-DTR mice (as in Fig. 4E), then let mice rest for 30 days. Next, we NAM-DTR mice with 550-L3 *Nb* larvae and let all mice to rest for another 33 days. We subsequently challenged WT and NAM-replenished mice with PR8 influenza (Fig. 4H). We found that mice with replaced NAMs following initial DT treatment lost significantly less weight following PR8 challenge compared to WT controls (Fig. 4I) and NAM-DTRs were completely protected from flu-induced morbidity compared to control (Fig. 4J). These findings further strengthen our hypothesis that *Nb-*induced training of NAMs represents a case of local innate immune training and that bone marrow derived replenished NAMs have similar functional capacity as the embryonic derived resident NAMs in response to influenza viral infection further validating our findings in Fig. 4A–C.

Next, we used WBP to assess lung function and fitness in all mice after PR8 challenge. We evaluated lung fitness among the three groups: NO-Tr, Tr-ON and Tr-OFF after PR8 infection, focusing on key parameters as described previously (Fig 1). We found that, following PR8 challenge the NO-Tr and Tr-OFF cohorts exhibited similar trends, including significant decrease in flow rate (*f*; Fig. 4K), a significant increase in mid-tidal expiratory flow (*EP50;* Fig. 4L), and a significant increase in time between breaths, indicating breathing difficulty (*Tp;* Fig. 4M). Additionally, both cohorts showed a significant increase in enhanced pause (*PenH*; Fig. 4N) and a significant decrease in relative peak expiratory flow rate (*Rpef*; Fig. 4O). In contrast, Tr-ON mice continued to maintain lung capacity metrics similar to those of uninfected controls (Fig. 1), while Tr-OFF mice, with bone marrow-replenished NAMs, provided mechanical lung properties akin to naïve mice. To further investigate, we euthanized the mice and collected total lung homogenates for infectious virus quantification and found no significant differences in the total infectious virus plaque-forming units among the groups (Fig. 4P). Histological analysis of lungs at day 6 post-PR8 challenge, using H&E staining (as in Extended Data Fig. 1C), revealed stark differences in lung infiltrates showing that NO-Tr and Tr-OFF airways are clogged compared to the Tr-ON airways (Fig. 4Q). We stained confocal lung sections from the three cohorts and analyzed them for neutrophils (Ly6G, red), NETs (H3, green) and NAMs (CD169) and found that both NO-Tr and Tr-OFF sections showed exuberant neutrophilia and NETs compared to Tr-ON, while no differences in the NAM proliferation following flu challenge highlighted by yellow arrow heads (Extended Data Fig. 5E). Our flow cytometry on the Tr-ON and Tr-OFF cohorts at 6dpi showed a significant decrease in the frequency of CCR3+ eosinophils (Fig. 4R) highlighting the critical association of NAMs and Eos along the large airways in trained mice. In summary, our data shows that the exclusive depletion of trained NAMs resulted in complete loss of protection. These findings indicate that trained NAMs are the primary cell-type exhibiting hallmarks of trained immunity and mediating protection against IAV infection in previously *Nb*-trained animals.

Our findings establish that trained NAMs are necessary for the host during PR8 infection, so we next asked if trained NAMs were also sufficient to rescue this protection from flu lethality. To do so, we developed an adoptive transfer system where we infected WT mice with *Nb* parasites as previously mentioned or injected them with PBS. On day 35, we purified naïve (from PBS treated) or trained NAMs using multiparametric flow cytometry sorting with purity of 95–98% pure NAMs (Fig. 4S). We used our NAM-DTR animals as hosts and depleted the NAM-niche by treating them with DT 2 days prior. We transferred 1×10^4^ naïve or trained NAMs intratracheally into NAM-DTR mice and challenged them with 75EID50 of PR8 virus, which is a sub-lethal dose in male mice 12–14h later. We then monitored weights for a week. We found that NAM-DTR hosts that received naïve NAMs lost tremendous weight compared to the NAM-DTR hosts that received trained NAMs (Fig 4T) despite no severe impact on mortality likely due to the sublethal dose. In summary, our data show that *Nb-*trained NAMs are necessary and sufficient to protect against flu challenge and hence play critical roles in the inflammation regulation and disease tolerance during heterologous infections.

### Eosinophils provide host protection against influenza-induced morbidity in trained mice.

**Extended Data Fig. 6:**
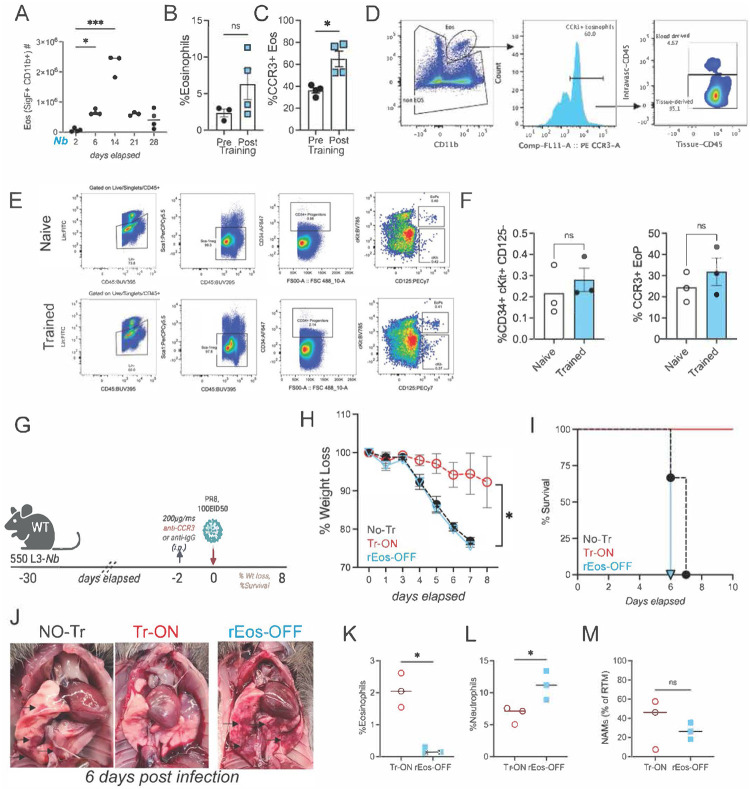
Eosinophils provide host protection against influenza-induced morbidity in trained mice. Flow-cytometry based (A) kinetics of eosinophils (Siglec-F+ CD11b+) showing total counts for four weeks after *Nb* infection. (B) Frequency of total eosinophils of total CD45+ cells and (C) Frequency of CCR3+ eosinophils of total eosinophils in naïve (d0, pre) and *Nb-*trained (d30, post) training. On the day of harvest, mice were injected with intravascular CD45-BUV661 3–4 minutes prior to euthanasia. Then lungs were harvested and stained and gated for eosinophils (SiglecF+ GR1- CD3- CD19- NK- MerTK- CD64- MHCII- CD11c-) (D) Flow gating strategy for (left) total eosinophils (Siglec-F+ CD11b+), (middle) CCR3+ eosinophils (right) intravascular CD45 (BUV661, y-axis) shown against total CD45 (PE-Cy7, x-axis). CCR3+ eosinophils were further gated and analyzed for distinguishing circulatory (intravascular CD45) from rEos (tissue embedded). (E) Flow graphs showing gating strategy of identifying eosinophil progenitors (EoP) in the bone marrow with naïve (top row) and trained (bottom row). Quantification showing (F) frequency of CD34+ c-Kit+ CD125- (EoP) and %CCR3+ EoP. WT mice were infected with 550-L3 *Nb* parasites and let to rest for 4 weeks, then treated with 200ug/mouse of anti-IgG or anti-CCR3 (i.p.) in 200ul. All mice were infected with 100EID50 PR8 (i.n.) 2 days later. (G) Experimental scheme (H) %Weight loss in PR8 infected WT mice (black circles and dotted black lines), or WT mice treated with anti-IgG (red circles with red line) or WT mice injected with anti-CCR3 (blue inverted triangles with blue lines). Data presented as Mean ± SEM, and statistical significance was determined by paired *t-*test (Wilcox test) and Mean±SEM. **p=*0.0078*. (I) Kaplan-Meier of %Survival curves for PR8 infected WT (black filled circles and dotted line), anti-IgG (red line) or anti-CCR3 treated (blue line with blue inverted triangles) mice. *n*=5/group; 3 independent experiments. Statistical significance was determined by Mantel-Cox test. **p=*0.0051*. (J) Representative images of gross pathology of NO-Tr, Tr-ON and rEos-OFF mice at 6 days post flu challenge. Flow cytometric evaluation on day 6 days post PR8 challenge of isotype (red open circles) or anti-CCR3 treated (blue filled squares) mice showing frequency represented of total CD45+ cells for (K) eosinophils or (L) Neutrophils and (M) NAMs presented as a frequency of RTMs. Data are shown as mean ± SEM. Statistical analysis done using *paired student’s t-test (Mann-whitney test),* n=3–4/group; ns = not significant, *p< 0.01, ****p<0.0001.

Our data clearly indicate that NAMs are indispensable for promoting disease tolerance during heterologous infection and that replacement of trained NAMs with untrained NAMs results in significant decrease in CCR3^+^ eosinophils (Fig. 4R); however, whether trained NAMs recruit and/or act in concert with other type 2 immune cells remains unclear. Following PR8 challenge, eosinophils increased selectively in trained mice and localized near large airways in close proximity to NAMs, where they served as a prominent source of IL-5. To determine whether this eosinophil phenotype was established before influenza challenge, we performed a time-course analysis after *Nb* training. Eosinophils rose over the first two weeks and returned to baseline by four weeks (Extended Data Fig. 6A. At the trained steady state (day 35), eosinophil frequency was similar in naïve and trained mice (Extended Data Fig. 6B), but eosinophils from trained lungs displayed ~two-fold higher CCR3 expression (Extended Data Fig. 6C). Intravenous anti-CD45 labeling further showed that ~95% of CCR3^+^ eosinophils were tissue-resident (rEos) rather than blood-derived (Extended Data Fig. 6D). To assess whether these CCR3^+^ rEos reflected increased bone marrow eosinophil output, we quantified eosinophil progenitors (EoP; Lin^−^ Sca1^−^ CD11b^−^ CD34^+^ c-Kit^+^ CD125^+^) and CCR3^+^ EoP in the bone marrow and observed no differences between naïve and trained mice (Extended Data Fig. 6F). Together, these data support a model in which *Nb* conditioning establishes a long-lived, tissue-resident CCR3^+^ eosinophil population that is present before influenza challenge and poised to collaborate with conditioned NAMs. Long-lived tissue-resident eosinophils (rEos) have been implicated as key regulators of lung homeostasis during type 2 inflammation^83–85^ and accordingly, anti-CCR3 antibody has been leveraged therapeutically to limit eosinophil responses and airway hypersensitivity in allergic asthma^86–88^. To test whether rEos contribute to protection from lethal influenza challenge, we depleted CCR3^+^ rEos in *Nb-*trained mice on day 33 and challenged mice 2 days later with high dose of influenza virus (Extended Data Fig. 6G; **hereafter “rEos-OFF”**). Compared with isotype-treated controls, rEos-OFF mice lost >20% body weight by day 6 post-infection and reached clinical endpoints (Extended Data Fig. 6H,I). Consistent with this, gross pathology revealed clear evidence of disease in untrained controls (No-Tr) and rEos-OFF mice, but not in trained mice (Tr-ON) (Extended Data Fig. 6J). Flow cytometry at day 6 post-challenge confirmed depletion specificity: anti-CCR3 treatment reduced eosinophils by ~70% (Extended Data Fig. 6K), was associated with a ~two-fold increase in neutrophils (Extended Data Fig. 6L) and did not alter NAM frequencies (Extended Data Fig. 6M). Together, these findings indicate that rEos are required to mitigate influenza-induced morbidity and suggest that trained NAMs may recruit and cooperate with rEos to maintain immune balance during severe respiratory viral infection.

#### Nb training induces chromatin reprogramming in NAMs that drives type 2 immunity, efferocytosis, and pro-resolving programs.

**Extended Data Fig. 7:**
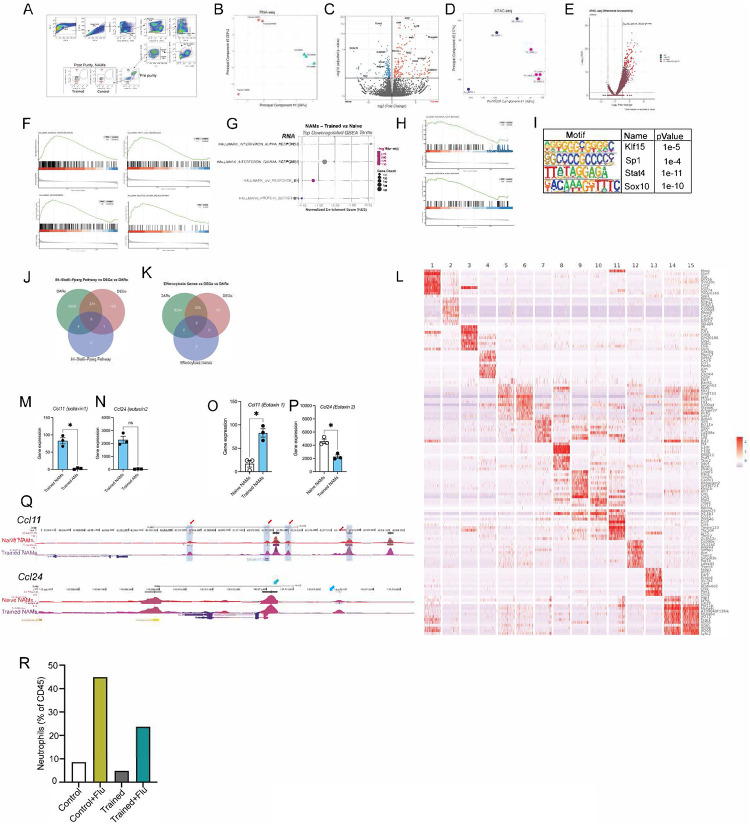
Distinct transcription on trained NAMs is driven by the differential gene transcription and chromatin accessibility suggesting epigenetic modification on NAMs (A) Flow gating strategy for sort purification showing pre and post sort purity for each sample. (B) PCA of bulk RNA-seq data showing distinct transcriptional profiles of naïve control NAMs (blue) and trained NAMs (red). (C) Volcano plot showing DEGs in trained NAMs (red) upregulated and (blue) downregulated compared to untrained naive NAMs with annotation of highly significant genes. (D) PCA of bulk ATAC-seq showing distinct chromatin accessibility profiles of naïve NAMs (blue) and trained NAMs (pink). One outlier replicate from the naïve NAM group was excluded from the analysis. (E) Volcano plot showing differential chromatin accessibility between trained and naïve NAMs based on bulk ATAC-seq analysis. The x-axis represents log_2_fold change in accessibility, and the y-axis shows −log_10_ adjusted p-value (FDR). Peaks with significantly increased accessibility in trained NAMs are shown in red on the right, while peaks with significantly decreased accessibility are shown in red on the left. Peaks with significant *p*-values but log_2_ fold change below the +/− 1 threshold are shown in blue, and peaks that do not meet significance or fold-change thresholds are shown in black. (F) GSEA plots illustrating representative upregulated Hallmark pathways in trained NAMs compared to naïve NAMs, including oxidative phosphorylation (top left), fatty acid metabolism (top right), adipogenesis (bottom left), and reactive oxygen species pathway (bottom right). For each pathway, the NES, adjusted p-value, and the number of leading-edge genes driving pathway enrichment are indicated. (G) Dot plot of GSEA showing the top downregulated Hallmark pathways (MSigDb) in NAMs from trained mice compared to naïve mice at day 35. The y-axis lists the names of the enriched pathway terms, while the x-axis shows the normalized enrichment score (NES) reflecting the magnitude of pathway enrichment. Dot size represents the number of genes contributing to each pathway, and dot color indicates the statistical significance of enrichment (−log_10_ adjusted p-value). (H)GSEA plots illustrating representative downregulated Hallmark pathways in trained NAMs compared to naïve NAMs, including interferon alpha response^52^ and interferon gamma response (bottom). For each pathway, the NES, adjusted p-value, and the number of leading-edge genes driving pathway enrichment are indicated. (I) Motif enrichment analysis performed using HOMER on the pooled set of leading-edge (core) genes derived from all significantly upregulated Hallmark pathways shown in (C). Using all genes with a cumulative TPM > 30 across all six samples as background, this analysis identified significant enrichment of *Klf15*, *Sp1*, *Stat4*, and *Sox10* transcription factors. Corresponding p-values for motif enrichment are shown.(J) Venn diagram showing the intersection between genes associated with DARs, DEGs, and the IL4-STAT6 pathway genes presented in Fig. 5E. (K) Venn diagram showing the intersection between genes associated with DARs, DEGs, and the efferocytosis genes presented in Fig. 5I. (L) KNeTL clustering generated heatmap of top 10 differentially expressed genes in the 15 individual immune clusters confirming their identity with red corresponding to higher expression. (M) Proportions of neutrophils from the Lin- CD45+ scRNA-seq analysis showing 8-fold increase in neutrophils in C+F (compared to C) resulting in lower resolution of other immune cells such as NAMs in that cluster. Bulk RNA-seq analysis of gene expression for naïve NAMs (white bars with clear circles) and naïve AMs (pink bars, clear squares) for (N) *Ccl11 (Eotaxin1) and* (O) *Ccl24 (Eotaxin2)*. Gene expression shown for naïve NAMs (white bars clear circles) and trained NAMs (blue bars filled circles) for (P) *Ccl11* and (Q) *Ccl24*. (R) IGV tracks from shown for bulk ATAC-seq on purified naïve NAMs (red histograms) and trained NAMs (purple histograms) for *Ccl11*^52^ and *Ccl24* (bottom). Red arrows showing the significant accessible peaks on NAM chromatin for respective gene loci and blue arrows pointing to accessible chromatin regions but not statistically significant between two conditions. Data presented as mean ± SEM, n=3. Representative tracks shown for each condition (n=4). Statistical analysis represented as student t test (Mann-Whittney test) *p=*0.05*.

**Figure-5:**
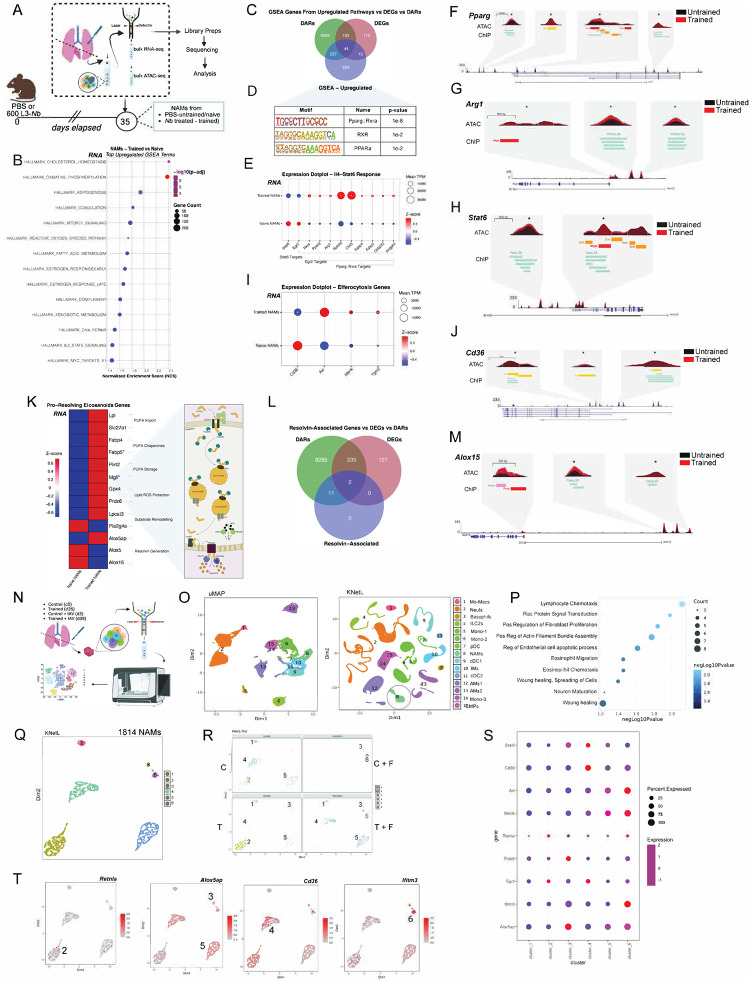
*Nb-*training induces a distinct chromatin remodeling and gene transcriptional program in NAMs priming them for enhanced type2, efferocytosis and wound repair programs. Mice were injected with PBS or 600 L3-stage *Nb* parasites and lungs were harvested on day 35, Liberase^™^ treated and stained for flow cytometry-based sort purification. From each parent sample, cells were distributed for bulk RNA-seq analysis (10K cells) and bulk ATAC-seq analysis (40K cells). Libraries were prepared according to Illumina protocols and subsequently sequenced for RNAseq and ATAC-seq analyses (n=3–4 per group). (A) Schematic representation of the experiment (B) Dot plot of Gene Set Enrichment Analysis (GSEA) showing the top upregulated Hallmark pathways (MSigDb) in NAMs from trained mice compared to naïve mice at day 35. The y-axis lists the names of the enriched pathway terms, while the x-axis shows the normalized enrichment score (NES)reflecting the magnitude of pathway enrichment. Dot size represents the number of genes contributing to each pathway, and dot color indicates the statistical significance of enrichment (−log_10_ adjusted p-value). (C) Venn diagram showing the intersection between genes associated with differentially accessible chromatin regions (DARs), differentially expressed genes (DEGs), and the pooled set of leading-edge (core) genes derived from all significantly upregulated Hallmark pathways (B). (D) Motif enrichment analysis performed using HOMER on the pooled set of leading-edge (core) genes derived from all significantly upregulated Hallmark pathways shown in (C). Using all genes with a cumulative TPM > 30 across all six samples as background, this analysis identified significant enrichment of nuclear receptor motifs, including PPARα and RXRA known motifs, as well as a PPARγ::RXRA de novo motif. Corresponding p-values for motif enrichment are shown. Dot plot showing TPM-normalized mRNA expression of selected IL-4-STAT6 pathway genes in naïve and trained NAMs. The y-axis indicates experimental condition, and the x-axis lists individual genes. Dot size reflects TPM expression levels, dot color represents z-score-scaled expression, and asterisk denote genes that are significant DEGs between conditions. (F, G, H, J, M) Genome browser tracks showing chromatin accessibility profiles from bulk ATAC-seq at the gene loci respectively. For each panel, ATAC-seq signal from a representative naïve NAM (dark gray) and trained NAM (red) is overlaid across the full gene locus and surrounding regulatory regions. Enlarged gray insets depict zoomed-in views of selected regions of interest. Asterisks indicate regions with significantly increased chromatin accessibility in trained NAMs as defined by DiffBind analysis. Annotated regulatory features are shown below each zoomed region, including promoter elements (red bars) and enhancer elements (yellow bars) based on ENCODE candidate cis-regulatory element (cCRE) annotations, as well as previously reported PPARγ binding sites derived from published macrophage ChIP-seq datasets, extracted via the UCSC Genome Browser ReMap ChIP-seq track (GEO accessions GSE63696, GSE92606, GSE111854, and GSE107456). Genome browser track showing chromatin accessibility profile from bulk ATAC-seq at the gene locus for (F) *Pparg*, (G) *Arg1*, (H) *Stat6,* (J) *Cd36* and (M) *Alox15*. (I) Dot plot showing TPM-normalized mRNA expression of selected efferocytosis genes in naïve and trained NAMs. The y-axis indicates experimental condition, and the x-axis lists individual genes. Dot size reflects TPM expression levels, dot color represents z-score-scaled expression, and asterisk denote genes that are significant DEGs between conditions. (K) Left: Heat-map showing TPM-normalized mRNA expression of genes associated with distinct stages of intracellular PUFA handling. Columns represent experimental conditions (naïve versus trained NAMs), and rows represent individual genes. Color intensity reflects z-score-scaled expression values, with red indicating relatively higher expression and blue indicating lower expression. Asterisks next to gene names denote genes that are significantly differentially expressed in trained NAMs. Right: Hypothetical graphical model illustrating how the genes shown in the heat map may contribute to sequential steps required for pro-resolving eicosanoid biosynthesis. PUFA such as arachidonic acid (AA) are transported via FABP4/5 transporters, then chaperoned to PLIN2 and further metabolized by MGLL. Processed PUFA are next released to phospholipases such as PLA2G4A and converted into mediators such as prostaglandins before finally synthesized by ALOX15 and secreted as pro-resolving lipids into the tissue for tissue-repair and anti-inflammatory functions. This schematic is intended as a conceptual framework to illustrate how trained NAMs may be primed for rapid activation of pro-resolving eicosanoid production upon secondary challenge, rather than a depiction of directly measured biochemical flux. (L) Venn diagram showing the intersection between genes associated with DARs, DEGs, and the genes included in the hypothetical pro-resolving eicosanoid production model. (N) Experimental schematic of single cell RNA seq analysis of lungs from (i) naïve or^106^ *Nb-*trained mice (d35 post *Nb*), or 3 days post PR8 infection of (iii) naïve or (iv) *Nb-*trained mice were stained for CD45^+^ and selected against Lineage markers (CD3, NK1.1 and B220), molecularly tagged and processed for scRNA-seq libraries using standard protocols. (O) uMAP (left) and KneTL (right) representations of 44,098 cells showing 15 unique clusters of innate immune cells presented as an aggregate of conditions i-iv. (P) Dot plot of GSEA showing the top upregulated Hallmark pathways (MSigDb) in NAMs from (cluster 8) compared to naïve mice at day 35. The y-axis lists the names of the enriched pathway terms, while the x-axis shows the negLog_10_ p-value. Size of the dot corresponds to number of genes represented and dot color indicates the statistical significance of enrichment (−log_10_ adjusted p-value). (Q) KNetL representations of re-clustered 1814 NAM genes (Cluster 8 from O) yielding six unique clusters from conditions i-iv shown as an aggregate. (R) NAM clusters are shown as dis-aggregated into individual conditions (i-iv as in M) with most prominent clusters shown in each condition. (R) De-aggregated NAM clusters from (P) for each condition. (S) Dot plot of individual NAM clusters (x-axis) showing enrichment of genes (y-axis) *Stat6, Cd36, Axl, Mertk, Retnla, Prdx6, Egr2, Ifitm3 and Alox5ap* as mentioned in ATAC-seq analysis. (T) Gene plots showing highly enriched genes for *Retnla* (cluster2), *Alox5ap* (cluster3 and cluster5), *Cd36* (cluster4) and *Ifitm3* (cluster6).

Collectively, these data indicate that Nb conditioning promotes survival through disease tolerance—enhancing type 2 immunity, restraining inflammation, and accelerating injury resolution—rather than through improved viral clearance. To define the transcriptional hallmarks of trained immunity in NAMs and infer their functional consequences, we flow-purified NAMs from naïve and trained mice and performed bulk RNA sequencing. (Fig. 5A, Extended Data Fig.7A).

Principal component analysis (PCA) of bulk RNA-seq data revealed distinct clustering of naïve (red circles) and trained NAMs (cyan triangles), indicating a transcriptional program unique to Nb training (Extended Data Fig. 7B). Differential gene expression analysis identified increased expression of immunoregulatory and tissue-repair genes in trained NAMs, including *Arg1, Chil3, Retnla, Spp1, Ly75, Cd9, Rnase2a and Cd200r2*, among others **(red**, Extended Data Fig. 7C), consistent with transcriptional programs that drive wound-repair responses in the lung following *Nb* infection^89–94^. An increased *Cd200r2* expression on CD206+ macrophages depends on IL-4 and IL-13 required to evade viral induced inflammation, which correlates with higher *CD200r2* on trained NAMs^95–97^. In parallel, PCA of bulk ATAC-seq profiles revealed distinct clustering of naïve (blue) and trained NAMs (pink), indicating a chromatin accessibility landscape unique to Nb training (Extended Data Fig. 7D). This analysis identified 24,456 regions that were significantly more accessible in trained NAMs. Peaks were assigned to genes using a ±3,000 bp window around transcription start sites (TSSs), yielding 8,534 associated genes (right, red; Extended Data Fig. 7E).

Gene set enrichment analysis (GSEA) of bulk RNA-seq data revealed enrichment of pathways including oxidative phosphorylation (OXPHOS), adipogenesis, fatty acid metabolism, DNA repair, and MTORC1 signaling (Fig. 5B, Extended Data Fig. 7F). These pathways predict an epigenetic and metabolic program in trained NAMs that supports disease tolerance through efficient efferocytosis, debris clearance, surfactant handling, and maintenance of lung homeostasis^4,98–100,101,102^. Enrichment of pathways related to Reactive Oxygen Species (ROS) and Xenobiotic Metabolism suggest predominant encoding of ROS-detoxifying and redox-buffering enzymes, including components of the glutathione-dependent antioxidant system (Fig. 5B, Extended Data Fig. 7F). This indicates enhanced capacity for handling oxidative stress and redox homeostasis rather than increased ROS production. In contrast, IFNα and IFNγ signaling pathways were downregulated in trained NAMs (Extended Data Fig. 7G, H) correlating inflammation reduction.

To connect these signatures to trained-immunity hallmarks, we focused on the GSEA leading-edge genes (LEGs). Integrating LEGs with differential expression (DEG) and differential accessibility (DAR) analyses showed that most LEGs were associated primarily with changes in chromatin accessibility rather than steady-state mRNA (Fig. 5C). Motif enrichment of the LEG set revealed strong enrichment for nuclear receptor motifs including RXRA/PPAR-family motifs and a de novo PPARγ:RXRA motif along with additional motifs (e.g., Klf15, Sp1, Stat4, Sox10) (Fig. 5D; Extended Data Fig. 7I), suggesting that trained NAM programs are organized around a PPAR/RXR-centered regulatory architecture. Notably, many of these genes are regulated by the IL-4–STAT6–PPARγ axis, a well-established driver of pro-resolving macrophage states that limits post-influenza fibrosis, restrains neutrophil persistence, and promotes clearance of inflammatory debris through efferocytosis and lipid metabolism. This pathway also supports wound repair and resolution via induction of Arg1 and Cd36 and reinforces anti-inflammatory signaling through IL-10^102,103,104,105^.

We next integrated the 510 GSEA leading-edge genes (LEGs) with differential expression (DEG) and differential accessibility (DAR) analyses and found that most LEGs were associated with changes in chromatin accessibility rather than steady-state mRNA (Fig. 5C). Motif enrichment^106^ across LEG-associated regulatory regions identified nuclear receptor motifs, including RXRA and PPAR-family motifs, as well as a de novo PPARγ:RXRA motif (Fig. 5D), together with additional motifs (Klf15, Sp1, Stat4, and Sox10; Extended Data Fig. 7I). Given the similarity among PPAR motifs and the known cooperation between PPARγ and RXRA, these data implicate PPARγ/RXR-centered regulatory elements as a key organizing feature of the training-associated LEG program.

We focused on three convergent programs—IL-4–STAT6–PPARγ signaling, efferocytosis, and pro-resolving lipid mediator pathways—which together provide an integrated framework linking metabolic rewiring, apoptotic cell clearance, and resolution biology^30^. Guided by our functional observations in trained NAMs and supported by the bulk transcriptomic and epigenomic data, we applied a locus-centered analysis to each program. For representative genes, we compared TPM-normalized mRNA expression and integrated ATAC-seq accessibility profiles with published macrophage PPARγ ChIP-seq datasets (ReMap/UCSC; GEO: GSE63696, GSE92606, GSE111854, GSE107456) and ENCODE candidate cis-regulatory elements (cCREs). In parallel, we quantified program overlap with differentially expressed genes (DEGs) and differentially accessible regions (DARs) using Venn analyses.

Among canonical IL4-STAT6 target genes, trained NAMs showed increased in expression of *Rxra*, *Pparg*, *Arg1*, *Chil3*, *Fabp5*, and *Angptl*, whereas *Stat6* and *Egr2* mRNA levels were reduced at this time point (Fig. 5E). Notably, irrespective of steady-state transcript abundance, all loci in Fig. 5E exhibited increased chromatin accessibility in trained NAMs, including multiple regions with significant differential accessibility (Extended Data Fig. 7J). At representative loci *(Pparg, Arg1, and Stat6),* accessibility was broadly increased across promoters and enhancers, with several annotated regulatory elements showing significant gains in accessibility (Fig. 5F, G, and H). For each gene examined, differentially accessible regions overlapped published macrophage PPARγ ChIP-seq peaks. Collectively, these data indicate that the IL-4–STAT6–PPARγ axis is epigenetically primed in trained NAMs. Next, we examined efferocytosis programs in trained NAMs. Efferocytosis is the receptor-mediated clearance of apoptotic neutrophils by macrophages, limiting secondary necrosis and promoting resolution and repair^104,107^. Among canonical efferocytosis receptors and execution factors, *Axl* and *Mertk* were expressed and trended toward higher mRNA levels in trained NAMs, although differences were not statistically significant at 35 days (Fig. 5I). In contrast, *Cd36* (a PPARγ-regulated scavenger receptor) was significantly reduced, whereas *Tgm2* (a PPARγ-regulated factor required for efficient efferocytosis)^108^, was significantly increased.

Importantly, irrespective of steady-state mRNA levels, all efferocytosis-associated genes in Fig. 5I showed increased chromatin accessibility in trained NAMs (Extended Data Fig. 7K). At the Cd36 locus, accessibility increased broadly across the gene body and flanking regulatory regions, including multiple ENCODE-annotated promoter and enhancer elements (Fig. 5J). These differentially accessible regions overlapped published macrophage PPARγ ChIP-seq peaks, indicating epigenetic priming despite reduced *Cd36* transcription at this late time point. Similar accessibility gains with overlap to PPARγ binding sites were observed at *Axl*, *Mertk*, and *Tgm2* (data not shown). Together, these data suggest that efferocytosis loci are epigenetically poised in trained NAMs at day 35, consistent with a primed state that can be rapidly engaged upon subsequent inflammatory challenge.

Tissue repair after infection is a core function of lung-resident macrophages, and given the regulatory, type 2–skewed phenotype of NAMs, we hypothesized that *Nb* conditioning primes NAMs for wound healing and resolution. We therefore examined pro-resolving eicosanoid pathways, as eicosanoids have been implicated in macrophage trained immunity^109^, pro-resolving eicosanoids including arachidonic acid–derived lipoxins and ω-3 polyunsaturated fatty acids (PUFA) derived resolvins promote inflammation resolution and tissue repair. Although individual lipid mediators cannot be inferred directly from transcriptomic or epigenomic data, their synthesis depends on coordinated regulation of PUFA uptake, trafficking, storage/remodeling, and enzymatic conversion. We therefore asked whether trained NAMs exhibit molecular signatures consistent with priming of pro-resolving eicosanoid biosynthesis.

TPM-normalized RNA-seq revealed coordinated upregulation of genes supporting multiple steps of PUFA handling and lipid mediator capacity in trained NAMs (Fig. 5K, left), including uptake (*Lpl, Slc27a1*), intracellular chaperoning (*Fabp4, Fabp5*), lipid droplet storage/mobilization (*Plin2, Mgll*), phospholipid remodeling (*Lpcat3*), and lipid peroxide detoxification (*Gpx4, Prdx6*). Most changes were modest, with Fabp5 and Mgll reaching significance. In contrast, several enzymes directly involved in pro-resolving eicosanoid synthesis (*Pla2g4a, Alox5, Alox15*) were reduced at this time point, while *Alox5ap* was increased. Together, these data suggest that trained NAMs preferentially upregulate lipid handling and redox-buffering modules, while enzymatic eicosanoid production remains transcriptionally restrained at baseline in the absence of active inflammation. Therefore, based on the signature of upregulated genes in the pro-resolving pathway, we deduce a hypothetical model (Fig. 5K, right) of lipid biosynthesis from uptake of PUFAs such as arachidonic acids to the synthesis of ALOX15 mediated secretion of pro-resolvins that may activate the Pparγ program thereby promoting anti-inflammatory macrophage phenotype in NAMs.

By day 35, mice have returned to a new homeostatic baseline without active infection, making constitutive production of pro-resolving eicosanoids unlikely. We therefore asked whether these pathways are epigenetically primed by assessing chromatin accessibility across genes in our PUFA-handling/pro-resolving model. Resolvin-associated genes preferentially overlapped with differentially accessible regions (DARs) rather than differentially expressed genes (DEGs), indicating predominant regulation at the chromatin level (Fig. 5L). Locus-level inspection supported this conclusion: at the Alox15 locus, trained NAMs showed broadly increased accessibility across the region, including at the promoter (not significant), and two distal regions with significantly increased accessibility that lay outside the annotated gene body (Fig. 5M). These distal DARs overlapped published macrophage PPARγ ChIP-seq peaks despite lacking ENCODE cCRE annotation, suggesting that priming at Alox15 is concentrated at distal PPARγ-associated regulatory elements. Similar accessibility gains—often at ENCODE-annotated regulatory regions and PPARγ-bound sites—were observed across the remaining genes in the model (data not shown).

Another hallmark of the heightened type 2 program in trained mice was the amplified eosinophil response after viral challenge (Fig. 2). To test whether NAMs contribute to eosinophil recruitment, we examined NAM expression of key eosinophil chemoattractants *Ccl11* and *Ccl24* (Eotaxin-1 and -2). Compared with alveolar macrophages (AMs) from trained mice, NAMs expressed significantly higher levels of Ccl11 and Ccl24 (Extended Data Fig. 7M,N), supporting NAMs—rather than AMs—as a major source of eosinophil-recruiting cues in the trained lung. Relative to untrained NAMs, trained NAMs showed a ~3-fold increase in Ccl11 transcripts and a modest decrease in *Ccl24* (Extended Data Fig. 7O,P), although *Ccl24* expression was already high at baseline in untrained NAMs. Consistent with these transcriptional changes, ATAC-seq revealed increased chromatin accessibility at the *Ccl11* locus in trained NAMs, with four regions exhibiting greater accessibility compared with untrained NAMs (Extended Data Fig. 7Q, **top**). In contrast, high accessibility across the Ccl24 locus was largely maintained in both naïve and trained NAMs (Extended Data Fig. 7Q, **bottom**).

Collectively, these data indicate that trained NAMs are transcriptionally restrained yet chromatin-poised across multiple steps required for pro-resolving eicosanoid generation at this late time point. We therefore propose that *Nb* training primes NAMs for rapid induction of pro-resolving lipid mediator biosynthesis upon secondary challenge, integrating PUFA uptake/handling, lipid remodeling, enzymatic conversion, and protection from lipid-associated oxidative stress (Fig. 5L). More broadly, these analyses reveal convergence across IL-4–STAT6–PPARγ signaling, efferocytosis, and pro-resolving lipid programs within a shared PPARγ-centered regulatory architecture: all 28 genes examined contained at least one nearby PPARγ ChIP-seq peak within the gene body or proximal regulatory regions, supporting a unifying role in coordinating primed resolution and repair programs in trained NAMs.

To determine whether influenza-exposed trained NAMs adopt distinct regulatory programs consistent with disease tolerance, we performed single-cell RNA-seq on flow-purified CD45^+^ Lin^−^ (CD3^−^ CD19^−^ NK1.1^−^) lung cells from four cohorts: naïve, *Nb*-trained at day 35 (restored homeostasis), PR8-infected controls, and *Nb*-trained mice at day 3 post-PR8 challenge (Fig. 5N). Samples (three biological replicates per group) were multiplexed by cell hashing. Across 44,098 cells, we resolved 15 innate immune clusters—including monocytes, Mo-Macs, neutrophils, basophils, ILC2s, DCs, IMs, AMs, NAMs, and CMPs—visualized by UMAP and KNetL (Fig. 5O). Consistent with prior reports^110,111^ eosinophils were not captured by standard scRNA-seq workflows. Cluster identities were defined using KNetL-based segregation and marker heatmaps (Extended Data Fig. 7L). Genes enriched in NAMs (1,052 total) were associated with immunoregulatory and repair-related pathways, including lymphocyte chemotaxis, Rac1-mediated efferocytosis, fibroblast proliferation, actin assembly, eosinophil recruitment, and wound repair (Fig. 5P).

We then extracted 1,814 NAMs across conditions and re-clustered them into six states (N1–N6) (Fig. 5Q), which redistributed across naïve (C), trained (T), infected control (C+F), and infected trained (T+F) groups (Fig. 5R; Extended Data Fig. 7R). N1 was broadly represented (reduced in C+F, consistent with the neutrophil-dominated composition) and expressed *Stat6*, *Cd36*, and *Prdx6* (Fig. 5S). N2 was enriched at trained steady state (T) and marked by Retnla (Fig. 5S,T), consistent with a reparative program. Following influenza, NAMs shifted toward infection-associated states: N3 was enriched in T+F and characterized by *Alox5ap* and *Prdx6*, whereas N6 dominated in C+F and showed high *Axl*, *Mertk*, and *Ifitm3* expression (Fig. 5R,S), consistent with heightened activation/efferocytosis-associated programs during severe inflammation. N4 was prominent in C and T+F and expressed *Cd36* and *Stat6*, while N5 co-expressed *Alox5ap* and *Mertk*, linking lipid-associated and efferocytosis modules (Fig. 5R–T). Together, these data indicate that *Nb* conditioning preserves a regulatory NAM baseline and enables rapid partitioning into specialized reparative, efferocytic, and lipid-associated states during influenza challenge—consistent with a trained, pro-resolving NAM program that supports disease tolerance.

### Trained NAM-mediated disease tolerance requires Arginase-1 and IL-4/IL-5–dependent lung conditioning

**Figure-6:**
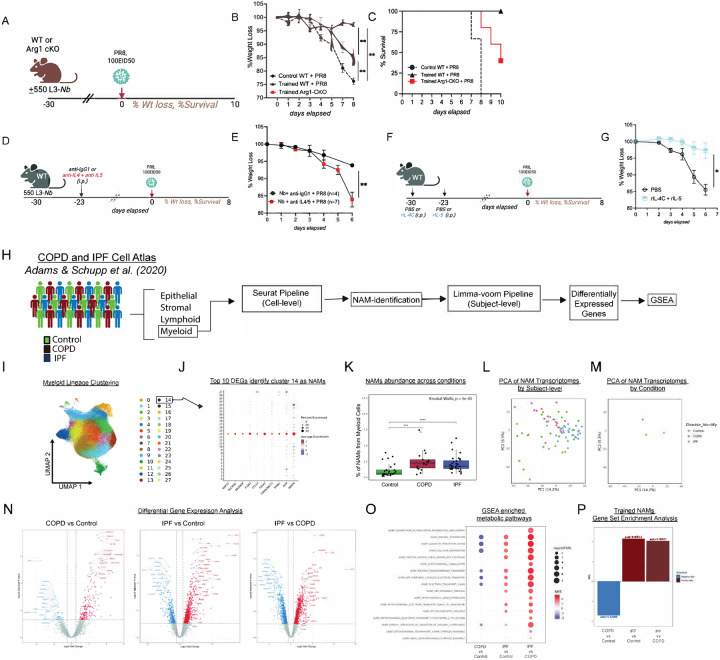
IPF NAMs align with trained, pro-resolving programs, whereas COPD NAMs diverge ^107^ WT or Arg1-CKO (*Cd169cre +/− x Arg1 flx/flx*) mice were infected with 550 L3-stage *Nb-*larvae (s.c.) and mice were let to rest for 30 days, then all mice, including a cohort without *Nb* treatment, were challenged with 100EID50 PR8 (i.n.) and mice were monitored for an additional 10d. (A) Experimental scheme (B) %Weight loss of mice control mice (WT, PR8 only; dotted black line with filled circles), Trained (WT, *Nb*+PR8; solid black lines with filled triangles) and Trained Arg-1 CKOs (red lines with filled squares) after PR8 challenge. Data shown in average weights represented as Mean ± SEM, *n*=*4/group*. Statistical significance was determined by paired *t-*test (Wilcox test) and Mean±SEM. **p=*0.005*. (C) of %Survival curves for untrained, trained and trained Arg-1 CKOs (as in B) following PR8 challenge. *n*=5/group; 2 independent experiments. Statistical significance was determined by Mantel-Cox test. (D,E) WT mice were infected with 550 L3-*Nb* larvae (s.c.) and let to rest for 7 days, then treated with either anti-IgG1 (200ug/mouse, i.p.) or anti-IL4/5 (200ug/each / mouse, i.p.) and let to rest for 3 weeks. Then all mice were challenged with 100EID50 PR8 (i.n.) and mice were monitored for extra 6d. (D) Experimental scheme (E) %Weight loss of trained WT mice treated with anti-IgG1(black lines with filled circles) or anti-IL4/5 treated (black lines with filled red squares) after PR8 challenge. Data shown in average weights represented as Mean ± SEM, *n*=*4/group*. Statistical significance was determined by paired *t-*test (Wilcox test) and Mean±SEM. **p=*0.005*. (E,F) WT mice were treated with PBS or recombinant IL-4C (rIL-4C), then let to rest for 7 days. On day 7, PBS treated mice were treated with PBS again and rIL4C (50ng/ms) treated mice were treated with rIL5 (50ng/ms). All mice were let to rest for another 3 weeks, then infected with 100EID50 PR8 (i.n.) and monitored for another 7 days. (F) Experimental scheme (G) %Weight loss of PBS treated mice (black lines with open circles) and rIL-4C/5 treated mice (blue lines with partially filled blue squares) after PR8 challenge. Data shown in average weights represented as Mean ± SEM, *n*=*4/group*. Statistical significance was determined by paired *t-*test (Wilcox test) and Mean±SEM. *p=*0.05*. (H) Schematic of analytical workflow, single-cell RNA-sequencing data from control, COPD and IPF lungs were processed through a Seurat-based pipeline to identify NAMs, aggregated at the subject level for limma–voom differential expression and subsequent gene set enrichment analysis (GSEA). (I) UMAP visualization of myeloid cells from the unsupervised clustering, with each point representing a single cell. (J) Dot plot of the top 10 differentially expressed genes for subcluster 14, highlighting the NAM-defining transcriptional signature. (K) Box plots showing the proportion of NAMs among all myeloid cells per subject in control, COPD and IPF lungs, with statistical comparisons by Kruskal–Wallis and pairwise significance determined by Wilcoxon rank-sum test with FDR correction: * p<0.05, ** p<0.01, *** p<0.001, **** p<0.0001. (L) Principal component analysis of NAM transcriptomes at the subject level, with each point representing the mean NAM expression profile for an individual subject colored by disease group. (M) Principal component analysis of NAM transcriptomes summarized by disease group, with each point representing the centroid of all subjects within that condition. **(N)** Differential gene expression in NAMs across disease conditions. Volcano plots showing log2 fold change and adjusted p-values from limma-voom differential expression analysis. Each point represents one gene, red points indicate significantly upregulated genes (log2FC > 0.25, FDR < 0.05); blue points indicate significantly downregulated genes (log2FC < −0.25, FDR < 0.05); grey points are not significant. Dashed lines indicate significance thresholds. (O) GSEA dot plot showing enriched metabolic pathway signatures. Color indicates normalized enrichment score (NES; blue = downregulated in disease, red = upregulated in disease); point size corresponds to −log10(FDR-adjusted p-value). (P) Trained NAM gene set enrichment across disease comparisons. Bar plot showing the NES for the trained NAM gene set in COPD vs Control, IPF vs Control, and IPF vs COPD. Red bars indicate positive NES (enrichment among upregulated genes), blue bars indicate negative NES (enrichment among downregulated genes). Annotated values above/below each bar show the FDR-adjusted p-value (padj) for the enrichment.

We asked which type 2–associated effector pathways are required for this trained state. Arginase-1 (Arg1) is a canonical IL-4–linked macrophage program that can promote tissue repair and restrain inflammation by competing with iNOS for arginine and limiting arginine-dependent inflammatory circuits^112–114^. In addition, resident tissue macrophages upregulate Arg1 after *Nb* infection to limit arginine availability required for juvenile parasite development in the lung^89^. Macrophage intrinsic Arginase-1 has been shown to suppress Th2-cytokine driven inflammation and fibrosis^112^ by converting arginine to ornithine, which promotes proline/hydroxyproline and polyamine biosynthesis in support of collagen deposition and proliferative tissue formation during wound repair^113^ as well as restraining inflammation by competing with iNOS for arginine and by lowering extracellular arginine availability and dampening the inflammatory NO-linked programs thereby limiting arginine-dependent T cell activation^114^. Consistent with this, our genomic analyses showed increased accessibility at the *Arg1* locus in trained NAMs (Fig. 5G) and elevated *Arg1* transcription (Extended Data Fig. 7C). To test whether macrophage-intrinsic Arg1 is required for trained NAM-mediated protection, we generated mice with conditional deletion of *Arg1* in CD169^+^ macrophages (*CD169-cre* x *Arg1*^*flx/flx*^:C57BL/6-*Arg1*^*tm1Pmu*^/J mice; “Arg1-CKO”) WT and Arg1-CKO mice were infected with *Nb*, rested for four weeks, and then challenged with PR8 (Fig. 6A). Whereas *Nb*-trained WT mice were fully protected from early weight loss, *Nb*-trained Arg1-CKO mice lost >15% body weight (Fig. 6B) and exhibited partial loss of survival benefit, with ~50% mortality by day 10 post challenge (Fig. 6C), compared with 0% mortality in trained WT mice and 100% mortality in untrained controls. These data identify macrophage-intrinsic Arg1 as a key mediator of trained NAM-dependent disease tolerance.

Because *Nb* infection elicits a robust type 2 immune response during the early conditioning phase—characterized by prominent induction of IL-4 and IL-5, we asked whether these cytokines are required to imprint NAM training and establish a protective lung state before influenza challenge. Thus, we neutralized IL-4 and IL-5 beginning on day 7 after *Nb* infection and challenged mice with PR8 after a three-week rest (Fig. 6D). Dual cytokine blockade significantly increased influenza-associated weight loss (Fig. 6E), indicating that IL-4/IL-5 signaling contributes to the conditioning phase required for protection. Conversely, to test whether type 2 cytokines are sufficient to phenocopy *Nb* conditioning, we administered rIL-4 complexes (rIL-4C) together with rIL-5 to WT mice 3–4 weeks before PR8 challenge (Fig. 6F). Compared with PBS controls, rIL-4C+rIL-5–treated mice were protected from early influenza-induced weight loss (Fig. 6G), demonstrating that type 2 cytokine conditioning can recapitulate key protective features of *Nb* training. Together, these results support a model in which *Nb*-driven IL-4/IL-5 conditioning imprints an Arg1-dependent NAM program that promotes disease tolerance during lethal influenza challenge, providing a mechanistic basis for leveraging type 2–conditioned macrophage states to mitigate severe inflammatory respiratory disease.

### IPF NAMs align with trained, pro-resolving programs, whereas COPD NAMs diverge

**Extended Data Fig. 8:**
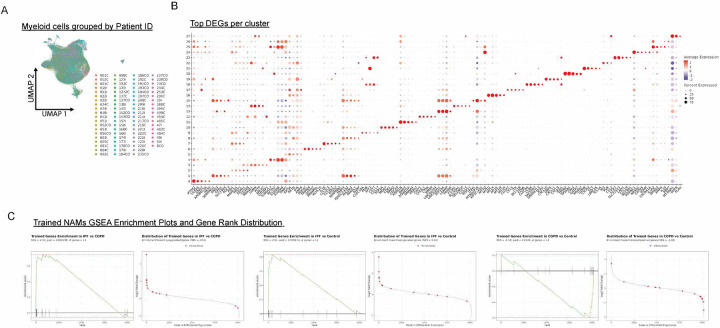
Single-Cell myeloid cell and trained NAM gene set enrichment across IPF, COPD and control patient cohorts. (A) UMAP of myeloid cell clustering, each point represents a single cell and color indicates patient identity, demonstrating thorough overlap across transcriptional space. (B) Dot plot displaying the top five most differentially expressed genes per myeloid cluster, ranked by log2 fold change. Dot size indicates percentage of cells expressing each gene and color reflects scaled average expression (blue = low, red = high). (C) Trained NAM gene set enrichment and rank distribution across disease comparisons. Gene set enrichment analysis (GSEA) using the trained NAM gene set in IPF vs COPD, IPF vs Control, and COPD vs Control. For each comparison, the left panel shows the enrichment curve of the human orthologs of the mouse genes upregulated on trained NAMs set across the ranked differential expression statistics (fgsea enrichment score; dashed line indicates maximum ES), with vertical ticks marking trained genes. The right panel shows the distribution of trained genes along the ranked log2 fold-change profile, with red points indicating trained genes.

To extend our findings to humans, we leveraged a published single-cell atlas^106^ to define NAM-like macrophages previously shown to be prominent in human lungs^15^ and asked how this population is transcriptionally remodeled in Chronic Obstructive Pulmonary Disease (COPD) (inflammatory pathology) versus Idiopathic Pulmonary Fibrosis (IPF) (exuberant repair/fibrosis), thereby testing whether the trained, pro-resolving NAM programs identified in our mouse model are conserved and disease-modulated in human lung disease. We annotated myeloid cells from a publicly available scRNA-seq atlas of patient cohorts of IPF and COPD patients (Fig. 6H). Unsupervised clustering analysis using a standard Seurat workflow identified 27 myeloid populations with distinct transcriptional profiles (Fig. 6I and Extended Data Fig.8A). NAMs identified based on the expression of canonical NAM markers *SLC40A1, FOLR2, F13A1, STAB1 and SELONOP* (Fig. 6J, Extended Data Fig. 8B). To determine whether the NAMs were differentially abundant across disease states, we quantified the frequency of NAMs as a percentage of total myeloid cells for each patient sample. NAM abundance varied across disease groups and pairwise comparisons revealed that NAM frequency was significantly elevated in IPF compared with controls. The enrichment of NAMs in these disease states suggested a dynamic interplay between NAMs and the disease pathophysiology of these conditions, prompting us to investigate whether NAMs also exhibited transcriptional differences across COPD and IPF. We aggregated NAMs to subject-level pseudo-bulk profiles and fitted limma-voom models with empirical Bayes moderation. PCA of voom-transformed expression showed separation of disease groups from controls along PC1 (14.2%) and PC2 (5.5%) (Fig. 6L). PCA of NAM-transcriptomes in these cohorts by condition showed a clear segregation pointing to nuances in differential NAM transcriptomes in control vs. COPD vs. IPF patients (Fig.6M).

DEG analysis was assessed for IPF versus Control, COPD versus Control, and IPF versus COPD. COPD-NAMs showed high transcription of cytoskeletal/migratory programs characterized by increased expression of *SRGAP2/2C/2B, ELMO1, NHSL1, WASHC2A* and *KLF13B* and downregulation of genes involved in phago-lysosomal acidification and vesicle trafficking such as *ATP6V1A, Atp6v0a1, VPS8, EXOC1* and *ADAP2* (Fig. 6N, left panel). IPF-NAMs exhibited a pronounced phenotypic shift towards a pro-wound healing phenotype characterized by downregulation of pro-inflammatory signaling nodes, *MALT1, LYN, MAPKAPK2, GRB2 a*nd upregulation of pro-fibrotic and lipid-handling genes such as *FN1, CCL8, SPP1, APOE, LPL, TREM2, CD9, MERTK, CD59 and FABP5* (Fig. 6N, middle panel, Extended Data Fig. 8C) akin to the pro-resolvin and efferocytosis NAM genes we found to be enriched in trained NAMs suggesting a strong correlation between our pro-resolvin and pro-efferocytosis signature with that found to be enriched in IPF NAMs (Fig 5I, L).

To identify coordinated transcriptional programs underlying the observed DEG profiles, GSEA using genes ranked by log2 fold change was performed and evaluated their Normalized Enrichment Scores (NES). Strikingly, NAMs from IPF lungs demonstrated robust enrichment of mitochondrial oxidative pathways relative to Control and COPD, including OXPHOS, aerobic respiration, electron transport chain activity, proton transmembrane transport, and ATP synthesis–coupled electron transport (Fig. 6O). This metabolic signature aligns with established paradigms linking OXPHOS to M2-like macrophage polarization, where mitochondrial metabolism supports anti-inflammatory and reparative functions, compared to glycolytic metabolism characteristic of pro-inflammatory M1-macrophages. In contrast, COPD-NAMs were downregulated for oxidative pathways relative to control and IPF NAMs, suggesting reduced mitochondrial activity and a possible metabolic shift towards glycolytic metabolism (Fig. 6O). Collectively, these findings indicate that IPF NAMs are metabolically rewired toward enhanced oxidative phosphorylation, whereas COPD-NAMs are skewed away from OXPHOS-dependent metabolism.

We next performed parallel analysis of the transcriptional programs enriched in human IPF-NAMs and COPD-NAMs in comparison to *Nb-*trained NAMs and mapped the top 100 most upregulated DEGs from bulk RNA-seq analysis of *Nb-*trained murine NAMs (Fig. 5), then compared them to their human orthologs for enrichment analysis. We found that the IPF-NAMs showed significant positive enrichment (IPF vs Control: NES = 2.15, padj = 1.58e-4; IPF vs COPD: NES = 2.05, padj = 1.87e-4), whereas COPD-NAMs were significantly negatively enriched for this gene set (COPD vs Control: NES = −1.68, padj = 0.0204), supporting activation of the trained NAM program in IPF and downregulation of such in COPD (Extended Data 8C and Fig. 6P).

Together, these analyses indicate that chronic lung disease differentially reprograms NAMs: IPF aligns with an OXPHOS-high, repair/lipid-handling state that overlaps with trained NAM programs, whereas COPD features a distinct state enriched for migratory/cytoskeletal modules and reduced oxidative metabolism. More broadly, they highlight the context dependence of reparative macrophage programs that are beneficial for resolving acute inflammatory injury, yet potentially maladaptive when sustained in fibrotic disease.

## DISCUSSION

We initiated this study early in the COVID-19 pandemic, when epidemiological data suggested that helminth-endemic regions showed milder disease severity^30,115–117^ hinting that the divergent immune response to COVID-19 pandemic is driven by personal immune history. The survival from the respiratory infections hinges on a balanced innate response and immunoregulatory pathways that prevent overwhelming inflammation and foster disease tolerance^118,119^. *Nb* conditioning rendered NAMs necessary and sufficient for protection from influenza lethality. Mechanistically, *Nb* training imprinted durable epigenetic and metabolic reprogramming in NAMs, enhancing immunoregulatory and repair-associated programs including Arginase-1 and pro-resolving lipid mediator pathways and promoting coordinated type 2 immunity, in part through recruitment and activation of eosinophils.

Human and preclinical studies suggest that trained immunity, which is defined as a metabolic and epigenetic reprogramming of innate cells can protect against respiratory viruses and other infections^22,27,29,30,52,120,121,22,122,123^, yet the underlying mechanisms remain unclear^120,124,125^. Previous studies describing heterologous infections have shown opposite effects in the degree of protection from secondary challenge^126–129^. One study showing heterologous model for respiratory infection builds on inoculation with a chronic parasitic infection such as *Heligmosomoides polygyrus* followed by RSV challenge, protection was associated with heightened type I interferon responses, which can be beneficial for RSV control resulting in lower virus^127^. However, excessive type I/III interferon signaling during influenza has been shown to exacerbate lung immunopathology and mortality^81^. Our model is distinct because we use a transient, lung-migratory helminth (*N. brasiliensis*) that is cleared from the lung within days and from the host by ~6–7 days, and we challenge >4 weeks later, long after parasite clearance.

Most prior studies of trained immunity emphasize “central” programming of bone marrow–derived myeloid cells, with limited evidence for durable, tissue-local macrophage training; one notable exception is an adenovirus model in which CD8^+^ T cell–derived IFN-γ activates alveolar macrophages and recruits neutrophils to protect against *Streptococcus pneumoniae*^130^. Progress in defining macrophage-specific mechanisms has also been constrained by nonselective depletion approaches such as clodronate liposomes^130,131^, which inadvertently affect monocytes and neutrophils^132,133^. Here, using a NAM-DTR model that enables selective NAM depletion, we demonstrate a distinct and previously unproven mechanism: *Nb* induces local trained immunity in NAMs that is both necessary and sufficient for survival after lethal influenza. Replacing trained NAMs with untrained NAMs abolished protection despite intact alveolar macrophages and other immune/stromal compartments, whereas adoptive transfer of trained NAMs restored protection. Thus, our study establishes tissue-resident NAM training as a direct driver of disease tolerance—limiting pathology without enhancing viral clearance.

To dissect NAM-intrinsic and -extrinsic mechanisms of protection, we found that *Nb* conditioning establishes a durable type 2–skewed milieu that persists through subsequent PR8 challenge—more than a month after the initial *Nb* infection—marked by increased IL-4, IL-5, IL-13, eotaxin-1, and IL-10. This sustained bias is consistent with a key feature of trained immunity: recalibrating inflammatory–regulatory balance to preserve pathogen control (type-1 response) while promoting disease tolerance. through an augmented type 2 response. After PR8 challenge, trained mice exhibited increased eosinophils and reduced neutrophils, with eosinophils as a major source of IL-5, a cytokine linked to tissue repair in the lunga^83,84,134–138,134^. Neutrophils, while critical for early immune responses and epithelial repair^139,140^, can also cause tissue damage through pro-inflammatory^69–72^ activity and formation of neutrophil extracellular traps (NETs)^141,142^.

We observed prominent neutrophil infiltration and NETs in control lungs but not trained lungs at day 6, coincident with greater tissue damage in controls. Functionally, disrupting type 2 conditioning during the *Nb* priming phase by neutralizing IL-4/IL-5 eliminated protection, whereas supplementation with recombinant IL-4 and IL-5 restored it. Together, these data support an essential role for type 2 cytokine–dependent lung conditioning in establishing protection, although whether IL-4/IL-5 act directly on NAMs remains to be determined. Our cytokine-conditioning experiments argue that a defined type 2 “training cassette” can substitute for helminth exposure: early IL-4 (with IL-5) is sufficient to imprint a long-lived, tissue-protective state that is recalled during influenza infection to limit immunopathology—potentially converting resident macrophages into “super healers.” Mechanistically, extensive prior work supports a direct IL-4→STAT6→PPARγ axis in macrophages that drives alternative activation and resolution biology: STAT6 promotes PPARγ activation and target engagement, and repeated IL-4 stimulation can progressively remodel macrophage regulatory circuitry in a PPARγ-dependent manner^108,143^. In parallel, IL-4 can induce PPARγ expression and endogenous lipid ligands through 12/15-lipoxygenase pathways, linking type 2 cues to CD36/efferocytosis and lipid-handling programs that are central to clearance of inflammatory debris and repair^144,145^. IL-5 is well established as a key survival/activation factor for eosinophils and increasingly appreciated as immunomodulatory contributor to tissue homeostasis providing a plausible route by which IL-5 driven eosinophil support reinforces the pro-repair niche (e.g. IL-5 availability, eotaxin guided positioning)^146^ that trained NAMs coordinate. Translationally, these findings motivate therapeutic strategies that deliver *controlled, localized* type 2 conditioning (e.g., long-acting IL-4 formulations such as IL-4 complexes with adjunct IL-5, or ex vivo IL-4/PPARγ-conditioned macrophage transfer) to program reparative, pro-resolving macrophage states without requiring helminth infection.

We found compelling evidence that *Nb*-training imprints durable epigenetic modification in NAMs consistent with hallmarks of macrophage memory. Integrating chromatin accessibility and transcriptional profiles in pseudo-bulk multiOme analyses identified the IL-4–STAT6–PPARγ axis as a central regulatory program linking three major features of trained NAMs: type 2 immunity, efferocytosis, and pro-resolving lipid pathways—highlighting a shared PPARγ-centered architecture. Among these targets, increased *Cd200r2* expression in trained NAMs is notable given evidence that IL-4/IL-13–dependent CD200R expression on CD206^+^ macrophages contribute to anti-inflammatory function^95,147^. For example, CD200:CD200R interaction has been shown to carry out immunoregulatory roles by “concerted, but opposing, activity of kinases and phosphatases”^96^. Our epigenetic studies showed greater accessibility of Arg1 among other genes in trained NAMs. Macrophage intrinsic Arginase-1 has been shown to suppress Th2-cytokine driven inflammation and fibrosis^112^ by converting arginine to ornithine, which promotes proline/hydroxyproline and polyamine biosynthesis in support of collagen deposition and proliferative tissue formation during wound repair^113^ as well as restraining inflammation by competing with iNOS for arginine and by lowering extracellular arginine availability and dampening the inflammatory NO-linked programs thereby limiting arginine-dependent T cell activation^114^. CD169 intrinsic conditional knockout of Arginase-1 resulted in partial loss of protection. The caveat of this, however, is that Arginase-1 is conditionally knocked out from all CD169+ macrophages, which encompasses both AMs and NAMs. Nevertheless, our data support a mechanistic role for arginase-1 (Arg1) as both an *output* and a *reinforcing effector* of the trained NAM state. Arg1 is a canonical IL-4–driven macrophage program induced via STAT6-dependent transcriptional control.

Our scRNA-seq analysis of 4 –way comparison of NAMs from naïve controls, trained and infected control and trained NAMs led us to identify subclusters of NAMs identifying *Retnla, Alox5ap, Cd36* and *Ifitm3* that define critical functions of NAMs during disease tolerance. While *Retnla* puts the brakes on tissue damage from excessive *Nb-*induced type 2 immunity and acts as an immunoregulatory gene, *Alox5ap* and *Alox12* regulates 12-HETE that facilitate activation, differentiation and tissue-remodeling of NAMs via secretion of pro-resolvins. Additionally, *Cd36* plays important roles in efferocytosis of dead neutrophils and NETs accelerating wound healing and *Ifitm3* regulates flu induced inflammation^148^ – all pointing to an elegant disease tolerance machinery driven by the Pparγ/IL-4/STAT6 chromatin program.

Our human meta-analysis further supports the principle that reparative macrophage logic is profoundly context dependent: programs that are protective when engaged transiently after acute injury can become maladaptive when chronically sustained. Consistent with this, NAM-like macrophages in IPF preferentially aligned with the trained, lipid-handling/efferocytosis-associated signatures we define in mice, whereas COPD NAMs diverged toward distinct, less reparative states—highlighting how the same core pathways can either support recovery or contribute to remodeling, depending on disease context. Across human and mouse studies, type 2–associated programs are repeatedly linked to reduced tissue damage and improved physiological outcomes—hallmarks of disease tolerance—including in SARS-CoV-2, where allergic/T2-high asthma endotypes have been associated with lower risk of severe disease in multiple cohorts^149–155^. Taken together, our study exhibits a local form of trained immunity in tissue-resident macrophages that is sufficient to protect the host from lethal type 1 viral immunopathology independent of adaptive immune enhancement, and it provides a conceptual framework for understanding how immune history sculpts divergent infection outcomes.

These findings nominate macrophage “conditioning” as a tractable strategy to promote disease tolerance—selectively engaging pro-resolving, reparative NAM programs to limit lung injury while preserving essential host defense. By defining a type-2-centered regulatory architecture that can be imprinted and recalled, this work opens a path to therapeutics that program “super healer” macrophages—through targeted cytokine conditioning, pathway-selective agonism, or cell-based approaches—to mitigate severe inflammatory respiratory infections and improve resilience to future viral pandemics, while also underscoring the need to tune intensity and duration to avoid maladaptive remodeling in chronic fibrotic disease.

## METHODS:

### STUDY DESIGN

Overarching goal of this study was to characterize innate memory of NAMs in heterologous infections as well as to investigate NAM intrinsic mechanisms that drive disease tolerance. To do so, we used a combination of genomic approaches, in situ microscopy, flow cytometry as well as functional assays in an in vivo murine system.

#### Mice

All mice used in this study were male and between the ages of 7 and 16 weeks at the start of the experiment. We used 3 main genotypes throughout the study: WT mice C57BL/6 were either purchased from Charles River Laboratories (C57BL/6NCr-NIH, 556) and left to be acclimated in the NYU vivarium for 2 weeks or were bred in-house in the NYU Vivarium; 2)NAM-DTR (*CD169-cre* mice crossed to *B6N.129P2-Cx3cr1*^*tm3 (DTR)Litt*^*/J* mice) (as described Ural et al., SI, 2020) were maintained on a C57BL/6 background and 3) Arg-1 CKO (*CD169-cre* mice crossed to C57BL/6-*Arg1*^*tm1Pmu*^/J mice) from purchased from Jackson Laboratories. An F1 cross generated *CD169cre x Arg1 fl* (homo/homo), further crossed to Arg1-fl to generate an experimental *CD169cre x Arg1-fl (het/homo)* were developed in house and maintained on B6 background and have been referred to as Arg-1 CKO in this study. Numbers of mice per experimental group are indicated in the figure legends. Throughout the study, all mice were maintained with food and water ad libitum under a 12-hour dark/light cycle, with 50–60% humidity and at 20–25 C in a pathogen free facility at New York University Langone Health Center. All experiments were performed with approval by the New York University Langone Health Center Institutional Animal Care and Use Committee and in accordance with guidelines from the National Institutes of Health, the Animal Welfare Act, and the U.S. Federal Law.

#### Parasite culture and inoculation of mice

*N. brasiliensis* L3 larvae were cultured and maintained as previously described^89^ in a petri dish culture containing 50% charcoal and 50% spaghnum peat moss. The larvae were isolated from cultures using a modified Baermann apparatus with 400U penicillin, 400 μg/ml each of Neomycin (GIBCO, Rockville, MD) and streptomycin in sterile 1X PBS, then washed with sterile PBS three times. Mice colonies were maintained by culturing fecal and cecal palettes from either STAT6^−/−^ or C57BL/6 mice. To maintain similar worm infectivity, L3 stage juvenile larvae were cultured from plates withing 8 weeks of preparation. For all experimental infections, mice were subcutaneously inoculated with 200μL suspension of 550–600 *Nb* L3 by lifting skin on the head between both ears under isoflurane anesthesia. For experimental consistency of infection based on worm motility, mice were infected with L3-larvae isolated from plates that had been prepared within 8 weeks to maintain the same degree of infectivity.

#### Influenza infection

Acute respiratory viral infection was introduced by an intranasal (i.n.) administration of influenza virus (H1N1/PR8) was purchased from Charles River Laboratories. Mice were infected with 75 to 150 EID50 (egg infectivity dose 50%) in 30μL suspension with sterile PBS through an intranasal route under anesthesia and analyzed in various time points as indicated in the main text.

#### Lung macroscopic analysis and histopathology.

Following euthanasia with CO2, mouse lungs were collected, fixed in 4% paraformaldehyde (PFA) for 72 hours at 4°C on a shaker. Fixed lungs were washed with PBS three times, then transferred into 70% ethanol, and paraffin-embedded for sectioning. Embedded lungs were sectioned at 5 μm and stained with hematoxylin and eosin (H&E). Lung damage was scored on 5 randomly selected high-power fields (40x) based on neutrophil infiltration and aggregation into lung parenchyma using previously described methods^29,156^.

#### Nuclei detection and quantification using QuPath

Image analysis for histochemical stains was conducted on QuPath (v0.6.0-x64, macOS) to quantify nuclear profiles. 40x images of histology were scanned and uploaded at the NYU core on Omero. High resolution images were imported into QuPath project and pixel calibration (um/pixel) was confirmed from metadata and /or a scale bar to enable area-based normalization. Tissue regions of interest (ROI) were defined using annotations to include tissue and exclude background and artifacts (folds/tears). For H&E slides, stain vectors were estimated, and nuclei were detected using QuPath’s watershed-based cell detection with the hematoxylin channel as the detection image; key parameters (threshold and min/max nuclear size constraints) were optimized on representative images and then applied uniformly across all images/conditions. Nuclei counts were computed as the number of detections within each ROI, and nuclei density was reported as total numbers within ROI. Detection overlays were visually inspected for quality control, and ROIs with major artifacts were refined or excluded before exporting final counts and densities.

#### Gross Pathology

Prior to harvest, lung images were collected using Samsung Galaxy S22+ and high-resolution images were uploaded on computer, then exported to illustrator for annotation and highlight disease and morphological features of PR8 infected lungs.

#### Tissue Preparation for Immunofluorescence and Confocal microscopy

Mice were euthanized using CO2 exposure and right lung lobes were harvested and subsequently fixed as previously described^3^. Tissues were fixed in paraformaldehyde (PFA), L-lysine, sodium periodate buffer (PLP, 0.05M phosphate buffer, 0.1M L-lysine, pH 7.4, 2 mg/mL NaIO4 and 40 mg/mL PFA) overnight at 4C on a shaker. Following day, tissues were dehydrated in 30% sucrose overnight at 4C and subsequently embedded in optimal cutting temperature compound (OCT) media. Frozen tissue sections were sectioned using Leica CM1850/Leica 3050S at a thickness of 20μm. Fc receptors were blocked with anti-CD16/32 Fc block antibody (BioLegend) diluted in PBS containing 2% serum and 2% FBS for 1 hour at room temperature. Fc receptor was blocked with anti-CD16/32 Fc block antibody (Clone: 93, Biolegend, San Diego, CA, USA) diluted in 1xphosphate buffer solution (PBS) containing 2% donkey or goat serum, 2% fetal bovine serum (FBS), and 0.01% Triton-X for 1 hour at room temperature. Sections were stained with Ly6G-BV421 or Ly6G-PE or Ly6G-BV510 (Clone: 1A8, Biolegend, San Diego, CA, USA), CD169-ef660 (Clone: Ser-4, Invitrogen, Hampton, NH, USA), Anti-Histone H3 (Abcam, Boston, MA, USA), Siglec-F-BV421 or Siglec-F AF488 or Siglec-F PE (Clone: E50–2440, BD Biosciences, Franklin Lakes, NJ, USA), IL-5-BV421 (Clone: TRFK5, Biolegend, San Diego, CA, USA), Keratin 5 (polyclonal, Biolegend) and SFTPC (polyclonal, Sigma) and diluted in blocking buffer for 1 hour at room temperature. Sections were then washed with 1xPBS and subsequently stained with Donkey anti-Goat 488 (ThermoFisher, Waltham, MA, USA), Goat anti-Rabbit 488 (ThermoFisher, Waltham, MA, USA), Goat Anti-Rabbit 546 (ThermoFisher, Waltham, MA, USA), Goat Anti-Mouse 488 (ThermoFisher, Waltham, MA, USA) in blocking buffer for 1 hour at room temperature and then washed with 1xPBS. After final wash, slides with sections were mounted using Immun-mount mounting medium (Fisher Scientific, Hampton, NH, USA) and Cover Glasses with a 0.13 to 0.17 mm thickness (Fisher Scientific, Hampton, NH, USA) were placed then left to dry overnight in drawers and slides were stored in dark at 4C until ready for microscopy. Fluorescence was detected with a Zeiss LSM 880 confocal microscope (Carl Zeiss, Oberkochen, Germany) equipped with 405, 488, 514, 561, 594, and 633 nm solid-state laser lines, a 32-channel spectral detector (409 to 695 nm), and 10×0.3, 20x Plan-Apochromat 0.8, 40x, and 63×1.40 objectives. Zen Black (Carl Zeiss, Oberkochen, Germany) software suite was used for data collection. Whole section imaging was imaged using an All-in-One Fluorescence Microscope BZ-X800 (Keyence Corporation) equipped with a eGFP, Cy3/TRITC, and Cy5 filter cube and a 20 × Plan Fluor Objective. Keyence images were stitched using BZ-X800 analyzer. Confocal imaging data were processed and analyzed using Imaris software version 8.3.1 (Bitplane USA; Oxford Instruments, Concord, MA, USA).

#### Tissue homogenization

Lungs were harvested at designated time points and washed in 1x PBS before placement in 2-mL tubes containing 500μL of sterile 1xPBS and one 2mm ceramic bead (MP Biomedical LLC). Lung tissue was homogenized with a FastPrep24 4.0M/s for 40s. After homogenization, lung debris was centrifuged at 15,000g for 5 min. Supernatant were aliquoted in 100uL aliquots and frozen down at −80C until used for analyses.

#### Preparation of cell suspensions for flow cytometry, and cell sorting

Single-cell suspensions of lung tissues were prepared for flow cytometry. Lung tissues were injected and incubated with RPMI 1640 (Lonza) media containing Liberase^™^ (5 mg/ml; Sigma-Aldrich for myeloid cell evaluation) or Collagenase I (100U/mL for lymphocyte evaluation) enzyme supplemented with 10% FBS (Gibco), 0.2% CaCl_2_, 0.2% MgCl_2_, deoxyribonuclease I, and 1% HGPG [1 mM HEPES, 5 mM l-glutamine, penicillin/streptomycin (10,000 U/ml), and gentamicin (5 μg/ml) (pH 7.5)] for 30 min at 37°C. Enzyme digestion was inactivated by addition of PBS containing 1 mM EDTA and 2% FBS. Lung tissue was dissociated into single-cell suspensions, and red blood lysis was performed using RBC lysis buffer (Alfa Aesar). Lung cells were resuspended in fluorescence-activated cell sorting (FACS) buffer [1× PBS (Gibco), 2% FBS, and 1mM EDTA]. Fc receptors were blocked with anti-CD16/32 Fc block antibody (BioLegend) and stained with the indicated antibodies (below) for 30 min at 4°C. Cells were fixed with 2% paraformaldehyde (PFA) for 10 min at RT and resuspended in FACS buffer. For intravascular (i.v.) staining to label blood vs tissue-resident immune cells, 1μg of anti-CD45 BUV661 (clone 30-F11, BioLegend) diluted in 150μL of PBS was injected retro-orbitally in mice under anesthesia. Mice were euthanized 3–4 minutes later, and organs were collected for analyses. Cell suspension was processed on Bio-Rad ZE5 instrument (Bio-Rad, Hercules, CA), and data analyzed using FlowJo software (BD Biosciences, Ashland, OR). For adoptive transfer experiments, 3 mice/each from PBS treated or *Nb-*infected mice were pooled after homogenization and prior to staining. Cells were stained with DAPI for continuous labeling of dead or dying cells in addition to CD45, Lin- dump channel (CD3, NK1.1, B220, CD31, EpCAM), CD11b, CD11c, MerTK, CD64 and Siglec-F. Cells were sorted using Symphony S6 SORP Sorter at NYU Flow Cytometry Core Facility. Post sort purity was checked for each sample and maintained at 95–98%. Aseptic conditions were maintained and sorted NAMs were washed twice to remove FBS using a swinging bucket centrifuge at 4C. Washed cells were reconstituted in sterile 1XPBS at 1×10^4^ cells per 80uL for intratracheal administration.

##### Antibody stains

Fc Receptors were blocked with anti-CD16/32 Fc block antibody (Clone: 93, Biolegend, San Diego, CA) and evaluation for lung myeloid cells was done by staining with Live Dead UV (AF350 NHS Ester, ThermoFisher, Waltham, MA), CD45-BUV395 (Clone: MEL-14, Biolegend, San Diego, CA), Siglec-F-BV421 (Clone: E50–2440, BD Bioscience, Franklin Lakes, NJ), MHCII-Pac Blue (Clone: M5/114.15.2, Biolegend, San Diego, CA), CX3CR1-BV605 (Clone: SA011F11, Biolegend, San Diego, CA), F4/80-BV650 (Clone: BM8, Biolegend, San Diego, CA), CD11b-BV711 (Clone: M1/70, BD Bioscience, Franklin Lakes, NJ), Ly6C-BV786 (Clone: HK1.4, BD Bioscience, Franklin Lakes, NJ), MerTK-PerCP-ef710 (Clone: DS5MMER, ThermoFisher, Waltham, MA), CD11c-PE (Clone: N418, ThermoFisher, Waltham, MA), CD64-PE Dazzle (Clone: X54–5/7.1, Biolegend, San Diego, CA), Ly6G-FITC (Clone: IA8, Biolegend, San Diego, CA), CD169-ef660 (Clone: Ser-4, ThermoFisher, Waltham, MA), CCR3-PE (Clone: J073E5, Biolegend, San Diego, CA). CD8+ specific H2-Db | Influenza A NP 366–374 | ASNENMETM (PE-labeled) or NP-Tet 311–325 (APC-labeled) were used for analysis of influenza specific CD8+ T cells 6 days post infection.

Stained cells were analyzed on a Bio-Rad ZE5 instrument with Everest Software^®^, and data were analyzed annotated using FlowJo^®^ software. For cell sorting, lung tissue was treated in the same way without fixation. After staining, cell pellet was directly resuspended in 1× PBS with 1 mM EDTA and 2% FBS. Cells were sorted on Becton Dickinson FACS Aria II instrument.

#### In vivo cell depletions

*In vivo* depletion of NAMs was done locally by intranasal administration of 15ng/mouse of DT twice 3 days apart or systemically by administration of a single i.p. injection of 40ng/g (200uL). Control mice were injected with 200μg of anti-IgG2b (clone LTF-2, BioXcell) as above. *In vivo* depletion of rEos (CCR3+ Siglec-F+ Ly6G- CD11b+ Ly6C-) in vivo, mice were injected i.p. with 200μg/mouse of anti-CCR3 (clone 6S2-19-4, BioXCell). *In vivo* depletion of IL-4 was done using anti-IL4 neutralizing antibody (Clone 11B11, BioXcell) along isotype control anti IgG2a (Clone RG7/1.30, BioXcell) and mice were treated with 200ug/mouse (i.p.) once on day 7 post *Nb* infection. *In vivo* depletion of IL-5 was done using anti-IL5 neutralizing antibody (Clone TRFK5, BioXcell) along isotype control anti IgG1 (Clone HRPN, BioXcell) and mice were treated with 200ug/mouse (i.p.) once on day 7 post *Nb* infection.

#### Adoptive Transfers and Challenge with PR8

For adoptive transfer experiments, 3 mice from each of the conditions of PBS treated or *Nb-*infected cohorts were harvested and digested with Liberase^™^ as described earlier. Single cell suspensions were stained for immune markers and subsequently sorted on Symphony S6 Sort Sorter. Post purity check, cells were resuspended in sterile 1XPBS after two washes, then reconstituted at a concentration of 1×10^4^ cells/ 80uL. Animals were anesthetized with ketamine + Xylazine, then mounted on slant plank and hung by their frontal teeth and administered 80ul of cell suspension intratracheally. Mice were monitored for additional 30 min to ensure waking up from anesthesia and no asphyxiation by cell transfer. 14h later, all mice were infected with 75EID50 (sublethal dose) intranasally and monitored for weight loss daily until end of study.

#### Cytokine and chemokine analysis

Supernatants from whole lung lysates were used for determination of chemokines and cytokines using Bio-Plex Pro mouse cytokine 23-plex Assay (Bio-Rad, M60009RDPD) or Mouse IFN-alpha/IFN-beta kit (ProcartaPlex^™^, EPX02A-22187–901) or viral titers.

#### Measurement of lung viral titers using Plaque Assay

Supernatants from whole lung homogenates were collected on 1-, 3- and 6-days post infection. Virus titers were determined on MDCK (Madin-Darby canine kidney) cells using a standard plaque assay. MDCK cells (1.5 × 10^6^ cells/cm^2^) were seeded in 6-well culture plates and incubated at 37°C in 5% CO2 for 24 hours. Cells were washed twice with 1× PBS before adding 200μl of a serial 10-fold dilution of mouse lung extracts [dilution prepared in 1× PBS and 0.3% bovine serum albumin (BSA)]. After 1 hour of incubation at 37°C, the inoculates were removed from the cells and overlay medium [2 ml per well; Dulbecco’s modified Eagle’s medium/F-12 Media; 2mM Glutamine, 10mM HEPES, 0.15% NaHCO3, 1% penicillin/streptomycin, 0.2% BSA, 0.6% Oxoid purified agar, 0.01% DEAE–dextran hydrochloride, and TPCK-trypsin (1 μg/ml)] was added to the cells, followed by incubation at 37°C in 5% CO2 for 72 hours. Subsequently, the cells were fixed with 4% paraformaldehyde in 1× PBS (1 mL per well) for at least 4 hours at room temperature. Plaque overlay was removed, and cells were washed with 1× PBS and set to dry overnight. Cells were then counterstained with 1% crystal violet for 1 min, washed briefly in water, and set to dry prior to plaque enumeration.

#### RNA extraction and RNA-seq analysis

Lung cell suspensions from 12–15 control C57BL/6 mice or *Nb*-trained mice were pooled for the RNA-seq analysis. Cells were obtained and sorted as indicated above. The RNAeasy plus micro kit (Qiagen, Valencia, CA) was used to extract total RNA from the sorted cells. RNA-seq libraries were prepared using the Illumina TruSeq Stranded mRNA Library Kit, and the individual libraries were pooled together. The samples were run over four lanes on the Illumina HiSeq2500 with 101 paired-end reads.

Sequencing reads were mapped to the reference genome (mm10) using the STAR aligner (v2.5.0c) (Dobin et al., 2013). Alignments were guided by a Gene Transfer Format (GTF) file. The mean read insert sizes and their standard deviations were calculated using Picard tools (v.1.126) (http://broadinstitute.github.io/picard). The read count tables were generated using HTSeq (v0.6.0) (Anders et al., 2015), normalized based on their library size factors using DEseq2 (Love et al. 2014), and differential expression analysis was performed. The Read Per Million (RPM) normalized BigWig files were generated using BEDTools (v2.17.0) (Quinlan and Hall, 2010) and bedGraphToBigWig tool (v4). Gene set enrichment analysis was performed using GSEA tool (PMID: 16199517). To compare the level of similarity among the samples and their replicates, we used two methods : principal-component analysis and Euclidean distance-based sample clustering. All the downstream statistical analyses and generating plots were performed in R environment (v3.1.1) (https://www.r-project.org/).

#### Motif Enrichment Analysis

To identify transcriptional regulators associated with the enriched pathways, transcription factor motif enrichment analysis was performed using HOMER (find.Motifs.pl)(v5.1). The foreground set consisted of 543 leading-edge (“core”) genes derived from significantly enriched, upregulated MSigDB Hallmark gene sets identified by GSEA. As a background, 14900 genes with cumulative expression of 30 TPM across six bulk RNA-seq samples were used to restrict the analysis to expressed genes. Motif enrichment was assessed in promoter regions spanning −300 bp upstream to +50 bp downstream of the annotated transcription start site, using default HOMER parameters and the mm10 mouse genome.

#### Single-cell RNA-seq sample prep and libraries

Mice were euthanized under CO2 and lung tissues were obtained from the control (naïve, n = 3), trained (*Nb* infected d35, n=3), control + PR8 (3dpi, n=3) and trained + PR8 (*Nb* trained + PR8 infected, d38, n=3) groups. Lungs were injected with 1mL of collagenase solution (as mentioned above) and incubated for 30 minutes at 37C incubator continuously shaking at 170rpm. Lungs were crushed using a 70um filter and lysed with red blood lysis buffer, then washed with 1XPBS substituted with 2% FBS and 2mM EDTA. Viable cells were counted for staining with trypan blue exclusion using Countess^®^ II (Life Technologies, AMQAX1000) and stained for viability, lineage markers (CD3, B220, NK1.1) all conjugated to PE-Dazzle and CD45-BV510. Equal number of stained cells from each animal were tagged with lipid oligo tags from Chromium Next GEM Single Cell 3ʹ v3.1: Cell Multiplexing kit (10X, 1000261). Cells were pooled into a single sample and sorted using Moflo XDP for sc-RNAseq. Cells were sorted using MoFlo cytometer (Beckman Coulter, VXX), and sorted cells were collected in 10% FBS supplemented sterile 1XPBS. Cells (10,500) were loaded per channel with an expected yield of 6000 cells. scRNA-seq libraries were prepared using the Single Cell 3’ Reagent Kits v2 (Chromium Single Cell 3’ Library and Gel Bead Kit v2, PN-120237), Single Cell 3’ Chip Kit v2 PN-120236 and i7 Multiplex Kit PN-120262″ (10x Genomics) (74), and the Single Cell 3’ Reagent Kits v2 user guide (manual part no. CG00052 Rev A). Libraries were run on a NovaSeq 6000. Alignment, barcode assignment, and UMI counting. The Cell Ranger Single Cell Software Suite (version 1.3) was used to perform sample demultiplexing, barcode ad unique molecular identifier^157^ processing, and single-cell 3′ gene counting.

#### Mouse: scRNA-sequencing and normalization

The single-cell count matrix was exported from 10x Genomics cell ranger version 3.0.0 output and subsequently analyzed in R version 3.4.1 Single Candle. Clustering and differential expression analysis were performed with the standard functions from Seurat version 2.3.4. Expression markers for each cluster were considered significant with an FDR of 5% and log_2_ FC as specified in Fig. 5.

#### Mouse: scRNA-sequencing data analysis

After confirming the integrity of the cDNA, quality of the libraries, number of cells sequenced and mean number of reads per cell, as a quality control, we used the CellRanger package to map the reads and generate gene-cell matrices. A quality control was then performed on the cells to calculate the number of genes, UMIs and the proportion of mitochondrial genes for each cell and the cells with low number of covered genes (gene-count < 500) and high mitochondrial counts (mt-genes > 0.2) were filtered out. Then the matrix was normalized based on their library sizes. A general statistical test was then performed to calculate gene dispersion, base mean and cell coverage to use to build a gene model for performing Principal Component Analysis (PCA) genes with high coverage (top 500) and high dispersion (dispersion > 1.5) were chosen (1990 genes) to perform PCA using iCellR R package (v1.5.5) (https://CRAN.R-project.org/package=iCellR). T-distributed Stochastic Neighbor Embedding (t-SNE), Uniform Manifold Approximation and Projection (UMAP), and K-nearest-neighbor-based Network graph drawing Layout (KNetL) were performed. KNetL map has a zoom option which allows the users to see more or less details (more or less sub-populations in cell communities). The network layout used in KNetL map is a force-based layout (Fruchterman and Reingold, 1991) and the zoom option is for changing the force in the system. Force-directed graph drawing algorithms assign attractive (analogous to spring force) and repulsive forces (usually described as analogous to the forces in atomic particles) to separate all pairs of nodes. (Fruchterman and Reingold, 1991). The network analysis that has been used in KNetL map have been long used for single cell analysis and clustering^158^, in here the nodes of the network layout are extracted and UMAP has been performed to create the final plot, a KNetL map. PhenoGraph^158^ clustering was then performed on the KNetL map results. Then the marker genes were found for each cluster and visualized on heatmaps, bar plots and box. The marker genes were then used to determine the cell types. Proportion (percentage) of the cell communities in each condition were calculated and Pseudotime Abstract KNetL maps (PAK map) were generated using iCellR.

#### Pathway and functional analysis

Gene Set Enrichment Analysis (GSEA), pathway and functional analysis were performed GSEA tool^159^. Before performing pathway-level analysis all genes tested for differential expression were ranked by the DESeq2 Wald statistic. Then the MSigDB Hallmark gene sets for *Mus Musculus* were obtained using the msigdbr package (v25.1.1). Finally, the clusterProfiler package (v4.16.0) was used to perform the GSEA analysis, where the pre-ranked list of genes tested for differential expression was analyzed against the MSigDB mice hallmark pathways. Gene sets of size 10–500 were included, and results were filtered using an FDR threshold of 0.25.

#### Human: scRNAseq analysis

Human single-cell RNA-sequencing data were obtained from the publicly accessible IPF Cell Atlas (Gene Expression Omnibus, accession GSE136831), comprising lung tissue biopsies from control donors and patients with chronic obstructive pulmonary disease (COPD) and idiopathic pulmonary fibrosis (IPF). Gene annotations (Ensembl gene IDs and symbols) were obtained using Ensembl BioMart (Ensembl Release 115, September 2025), querying the Human genes (GRCh38.p14) dataset and retrieving the “Gene stable ID” and “Gene name” attributes.

##### Analysis Packages used

All analyses were performed in R (4.4.2) using Seurat (5.4.0), edgeR (4.4.2), limma (3.62.2), fgsea (1.32.4), clusterProfiler (4.14.6), msigdbr (25.1.1), enrichplot (1.26.6), dplyr (1.1.4), tidyr (1.3.2), tibble (3.2.0), ggplot2 (3.5.1), ggpubr (0.6.2), ggrepel (0.9.6), patchwork (1.3.2), and RColorBrewer (1.1.3).

Data were subsetted to include only myeloid-annotated cells. The myeloid subset was processed in R with Seurat’s standard workflow, applying NormalizeData(), FindVariableFeatures (vst; n=2,000), ScaleData() with regression of the “Library_Identity” covariate, PCA(), FindNeighbors(), FindClusters(), FindSubCluster(), and RunUMAP(). Cluster marker genes were identified using Seurat’s FindAllMarkers (), restricting results to positively enriched markers in at least 25% of cells exhibiting a log2 fold-change >0.25; P values were corrected for multiple testing using the Benjamini–Hochberg method, and significance was defined as adjusted P<0.05.

Per-subject NAM frequency was calculated as 100 × (NAMs / Total myeloid cells). Differences in NAM abundance among Control, COPD, and IPF were evaluated using a Kruskal–Wallis test followed by pairwise Wilcoxon rank-sum tests with Benjamini–Hochberg FDR correction.To compare NAM transcription across disease states and avoid pseudo-replication from cell-level testing, we performed subject-aware differential expression analysis. NAM cells were aggregated per subject using Seurat’s AggregateExpression() yielding a gene-by-subject count matrix aligned to metadata (Subject_Identity, Disease_Identity). Lowly expressed genes were filtered prior to modeling, and library-size effects were accounted for using the limma–voom workflow to obtain log2-CPM values with precision weights. A linear model was fit with a design matrix encoding Control, COPD, and IPF groups, and contrasts were specified for IPF vs Control, COPD vs Control, and IPF vs COPD. Empirical Bayes moderation was applied, and multiple testing correction used Benjamini–Hochberg, with differentially expressed genes defined at FDR<0.05. PCA was performed on the subject-level pseudobulk (voom-transformed log2-CPM) expression matrix; genes were centered and scaled to unit variance prior to PCA, and variance explained by PC1–PC2 was reported. Samples were colored by Disease_Identity (Control, COPD, IPF) and plotted in PC1–PC2 space with axis labels indicating the percentage of variance explained by each component; axis limits were fixed to the full range of the data to ensure comparable visualization across panels. Group centroids were computed as the mean PC1 and PC2 coordinates for each disease category and visualized as diamonds to summarize separation among groups.

Gene Set Enrichment Analysis (GSEA) of Gene Ontology Biological Process gene sets was performed on the subject-aware differential expression results for NAMs across the three contrasts (IPF vs Control, COPD vs Control, IPF vs COPD). For each contrast, genes were pre-ranked by the descending log2 fold-change, with genes lacking statistics removed and, where multiple entries mapped to the same symbol, the instance with the largest absolute log2 fold-change retained. Enrichment was assessed with Benjamini–Hochberg adjustment, p-value cutoff 0.05, and gene set size bounds of 10–500. In addition, enrichment of our murine NAM Trained gene set was evaluated across the three contrasts. Cross-species gene symbol mapping was performed using Ensembl BioMart (Ensembl Release 115, September 2025), querying the Mouse genes (GRCm39) dataset and retrieving Human Orthologues attributes corresponding to human GRCh38.p14. Following conversion of the murine Trained NAM genes to their human orthologs (Human gene name; HGNC symbols), fgsea was run with 10,000 permutations and gene set size bounds of 10–500; significance was assessed using Benjamini–Hochberg–adjusted p-values (padj < 0.05).

Thresholds for patients were determined as such:

IPF: median 1.74% [IQR 1.23%; range 0.56–6.16%], n=32; Control: median 0.65% [IQR 0.74%; range 0.19–3.87%], n=27; Mann-Whitney U test, FDR-adjusted p = 9.1×10^−5).

COPD samples as well had an increase in NAM abundance relative to controls (COPD: median 2.24% [IQR 1.31%; range 0.45–7.46%], n=17; vs Control: median 0.65% [IQR 0.74%; range 0.19–3.87%], n=27; Mann-Whitney U test, FDR-adjusted p = 5.7×10^−4) (Fig. 6K). No significant differences were observed between IPF and COPD (FDR-adjusted p = 0.399).

#### ATAC-seq sample prep and libraries

A total of 50,000 purified macrophage populations per replicate were washed once in cold sterile PBS and resuspended in 20–50 μl of cold lysis buffer (10 mM Tris-HCl (pH 7.4), 10 mM NaCl, 3 mM MgCl_2_ and 0.1% IGEPAL CA-630). Cell lysates were centrifuged at 500*g* for 30 seconds to 1 min at 4 °C, and nuclei were resuspended in 25 μl of water. The transposition reaction mix consisted of 25 μl of TD buffer, 2.5 μl of Tn5 transposase (Illumina cat. 20034198), and 22.5 μl of nuclease-free water containing the nuclei. The mix was incubated for 30 min at 37 °C. Transposed DNA fragments were purified using a Qiagen Reaction MiniElute kit (Qiagen). Clean DNA was run with PCR amplification to incorporate the indexes, which were a universal index and a unique i7 index per sample. (IDT primers outlined in Buenrostro 2013 paper) and amplified by PCR for 11 cycles using NEBNext high fidelity 2× PCR master mix (New England Biolabs). PCR products were cleaned with 0.9x volume of Ampure XP beads (Beckman Coulter), and amplified fragment size was verified on an Agilent 2100 Bioanalyzer system (Agilent) with high sensitivity DNA screentape. ATAC-seq libraries were quantified by quantitative PCR with the Roche-kapa Illumina quantification kit. Paired-end sequencing was performed on an Illumina Novaseq6000 system with an SP100 cycle kit sequenced as paired-end 50 bases.

#### ATAC-seq pre-processing & analysis

Preprocessing of reads was done automatically by seq2science (v1.2.4) using the atac-seq workflow. Paired-end reads were trimmed with fastp (v0.23.2) with default options. Genome assembly mm10 was downloaded with genomepy (v0.16.3). Reads were aligned with bwa-mem2 (v2.2.1) with options ‘-M’. Afterwards, duplicate reads were marked with Picard MarkDuplicates (v3.0.0). General alignment statistics were collected by samtools stats (v1.16). Before peak calling, paired-end info from reads was removed with seq2science so that both mates in a pair get used. Peaks were called with macs2 (v2.2.7) with options ‘--shift −100 --extsize 200 --nomodel --buffer-size 10000’ in BAM mode. The effective genome size was estimated by khmer (v3.0) by taking the number of unique k-mers in the assembly of the same length as the average read length for each sample. The UCSC genome browser was used to visualize and inspect alignment. Quality control metrics were aggregated by MultiQC (v1.14).

For differential accessibility analysis per-sample MACS2 peak sets were provided to DiffBind (v3.18.0), which constructed a consensus peak set by merging overlapping peaks across samples. Read counts were generated over the consensus peak regions and normalized using DiffBind default settings. Differential chromatin accessibility was assessed using DiffBind with DESeq2 as the statistical framework, and differentially accessible regions were identified at an adjusted p-value (FDR) threshold of 0.05. Significant regions were subsequently annotated to nearby genes using ChIPseeker (annotatePeak)(v1.44.0) with the TxDb.Mmusculus.UCSC.mm10.knownGene (v3.10.0) transcript database, using a +/− 3kb window around the transcription start sites. Gene identifiers were mapped to gene symbols using org.Mm.eg.db (v3.21.0). Annotated regions were classified as promoter-associated peaks or distal/enhancer-like peaks (Distal, Intergenic, or Intron), and unique gene symbols from each category were extracted for downstream analyses

#### Whole body plethysmography analysis

Plethysmography tests were performed using previously established protocols^48^ with necessary modifications for this study. Naïve, trained or flu-infected mice were introduced to a plethysmography chamber (Buxco Systems, Wilmington, NC) which was placed in an unlit biological safety cabinet and mice were acclimated to the chamber for 10 minutes. Following acclimation, mice were actively measured for a 10-minute period. Mice were randomly assigned to plethysmography chamber for intended recordings. During the 10-minute measurement period within the plethysmograph, the plethysmograph continually measured respiratory responses, with 2-second summaries for 11 various respiratory parameters such as *f, Tdv, Mvb, Penh, Rpef, PIF, PEF, Ti, Te, EF50 and Tr*. For each condition, we used 4–5 animals and computed the geometric mean presented as ± SEM. For each parameter, calculations were made by the *Finepointe* software (v2.9.0) and exported on excel for generating data graphs. Data shown in Fig. 1 and 4 only for selected members chosen as described in other published studies with differences in - *f, PenH, Tp, Ep50, Rpef*.

## Extended Data

## References

[1] Hartmann W., Brunn M. L., Stetter N., Gabriel G. & Breloer M. Pre-existing helminth infection impairs the efficacy of adjuvanted influenza vaccination in mice. PLoS One 17, e0266456 (2022). 10.1371/journal.pone.0266456 35358281 PMC8970517

[2] Fels A. O. & Cohn Z. A. The alveolar macrophage. J Appl Physiol (1985) 60, 353–369 (1986). 10.1152/jappl.1986.60.2.353 3005225

[3] Ural B. B. Identification of a nerve-associated, lung-resident interstitial macrophage subset with distinct localization and immunoregulatory properties. Sci Immunol 5 (2020). 10.1126/sciimmunol.aax8756 PMC7717505 32220976

[4] Zuttion M. Interstitial Macrophages Mediate Efferocytosis of Alveolar Epithelium during Influenza Infection. Am J Respir Cell Mol Biol 70, 159–164 (2024). 10.1165/rcmb.2023-0217MA 38207122 PMC10914771

[5] Carey B. & Trapnell B. C. The molecular basis of pulmonary alveolar proteinosis. Clin Immunol 135, 223–235 (2010). 10.1016/j.clim.2010.02.017 20338813 PMC2866141

[6] Schyns J. Non-classical tissue monocytes and two functionally distinct populations of interstitial macrophages populate the mouse lung. Nat Commun 10, 3964 (2019). 10.1038/s41467-019-11843-0 31481690 PMC6722135

[7] Neupane A. S. Patrolling Alveolar Macrophages Conceal Bacteria from the Immune System to Maintain Homeostasis. Cell 183, 110–125 e111 (2020). 10.1016/j.cell.2020.08.020 32888431

[8] Maus U. A. Role of resident alveolar macrophages in leukocyte traffic into the alveolar air space of intact mice. Am J Physiol Lung Cell Mol Physiol 282, L1245–1252 (2002). 10.1152/ajplung.00453.2001 12003780

[9] Gautier E. L. Systemic analysis of PPARgamma in mouse macrophage populations reveals marked diversity in expression with critical roles in resolution of inflammation and airway immunity. J Immunol 189, 2614–2624 (2012). 10.4049/jimmunol.1200495 22855714 PMC3537497

[10] Gautier E. L. & Yvan-Charvet L. Understanding macrophage diversity at the ontogenic and transcriptomic levels. Immunol Rev 262, 85–95 (2014). 10.1111/imr.12231 25319329

[11] van de Laar L. Yolk Sac Macrophages, Fetal Liver, and Adult Monocytes Can Colonize an Empty Niche and Develop into Functional Tissue-Resident Macrophages. Immunity 44, 755–768 (2016). 10.1016/j.immuni.2016.02.017 26992565

[12] Gibbings S. L. Three Unique Interstitial Macrophages in the Murine Lung at Steady State. Am J Respir Cell Mol Biol 57, 66–76 (2017). 10.1165/rcmb.2016-0361OC 28257233 PMC5516280

[13] Chakarov S. Two distinct interstitial macrophage populations coexist across tissues in specific subtissular niches. Science (New York, N.Y.) 363 (2019). 10.1126/science.aau0964 30872492

[14] Li X. Coordinated chemokine expression defines macrophage subsets across tissues. Nature immunology 25, 1110–1122 (2024). 10.1038/s41590-024-01826-9 38698086 PMC11565582

[15] Yeung S. T. Nerve- and airway-associated interstitial macrophages mitigate SARS-CoV-2 pathogenesis via type I interferon signaling. Immunity 58, 1327–1342 e1325 (2025). 10.1016/j.immuni.2025.04.001 40286790 PMC12096317

[16] Netea M. G. Training innate immunity: the changing concept of immunological memory in innate host defence. Eur J Clin Invest 43, 881–884 (2013). 10.1111/eci.12132 23869409

[17] Netea M. G. Defining trained immunity and its role in health and disease. Nat Rev Immunol 20, 375–388 (2020). 10.1038/s41577-020-0285-6 32132681 PMC7186935

[18] Netea M. G., Quintin J. & van der Meer J. W. Trained immunity: a memory for innate host defense. Cell Host Microbe 9, 355–361 (2011). 10.1016/j.chom.2011.04.006 21575907

[19] Ochando J., Mulder W. J. M., Madsen J. C., Netea M. G. & Duivenvoorden R. Trained immunity - basic concepts and contributions to immunopathology. Nat Rev Nephrol 19, 23–37 (2023). 10.1038/s41581-022-00633-5 36253509 PMC9575643

[20] Divangahi M. Trained immunity, tolerance, priming and differentiation: distinct immunological processes. Nature immunology 22, 2–6 (2021). 10.1038/s41590-020-00845-6 33293712 PMC8020292

[21] Johansson A. Trained immunity and epigenetic memory in long-term self-renewing hematopoietic cells. Experimental hematology 121, 6–11 (2023). 10.1016/j.exphem.2023.02.001 36764598

[22] Moorlag S. beta-Glucan Induces Protective Trained Immunity against Mycobacterium tuberculosis Infection: A Key Role for IL-1. Cell Rep 31, 107634 (2020). 10.1016/j.celrep.2020.107634 32433977 PMC7242907

[23] Riksen N. P. & Netea M. G. Immunometabolic control of trained immunity. Mol Aspects Med 77, 100897 (2021). 10.1016/j.mam.2020.100897 32891423 PMC7466946

[24] Tran K. A. BCG immunization induces CX3CR1(hi) effector memory T cells to provide cross-protection via IFN-gamma-mediated trained immunity. Nature immunology 25, 418–431 (2024). 10.1038/s41590-023-01739-z 38225437

[25] Domínguez-Andrés J. Trained immunity: adaptation within innate immune mechanisms. Physiological reviews 103, 313–346 (2023). 10.1152/physrev.00031.2021 35981301

[26] Giamarellos-Bourboulis E. J. Activate: Randomized Clinical Trial of BCG Vaccination against Infection in the Elderly. Cell 183, 315–323.e319 (2020). 10.1016/j.cell.2020.08.051 32941801 PMC7462457

[27] Kaufmann E. BCG vaccination provides protection against IAV but not SARS-CoV-2. Cell Rep 38, 110502 (2022). 10.1016/j.celrep.2022.110502 35235831 PMC8858710

[28] Prentice S. BCG-induced non-specific effects on heterologous infectious disease in Ugandan neonates: an investigator-blind randomised controlled trial. The Lancet. Infectious diseases 21, 993–1003 (2021). 10.1016/s1473-3099(20)30653-8 33609457 PMC8222005

[29] Khan N. β-Glucan reprograms neutrophils to promote disease tolerance against influenza A virus. Nature immunology (2025). 10.1038/s41590-024-02041-2 PMC11785525 39779870

[30] Saeed S. Epigenetic programming of monocyte-to-macrophage differentiation and trained innate immunity. Science (New York, N.Y.) 345, 1251086 (2014). 10.1126/science.1251086 25258085 PMC4242194

[31] Afkhami S. Respiratory mucosal delivery of next-generation COVID-19 vaccine provides robust protection against both ancestral and variant strains of SARS-CoV-2. Cell 185, 896–915 e819 (2022). 10.1016/j.cell.2022.02.005 35180381 PMC8825346

[32] Lercher A. Antiviral innate immune memory in alveolar macrophages following SARS-CoV-2 infection ameliorates secondary influenza A virus disease. Immunity (2024). 10.1016/j.immuni.2024.08.018 PMC11563926 39353439

[33] Ngo V. L. Intestinal microbiota programming of alveolar macrophages influences severity of respiratory viral infection. bioRxiv (2024). 10.1101/2023.09.21.558814 PMC10942762 38295788

[34] Aegerter H. Influenza-induced monocyte-derived alveolar macrophages confer prolonged antibacterial protection. Nature immunology 21, 145–157 (2020). 10.1038/s41590-019-0568-x 31932810 PMC6983324

[35] Medzhitov R., Schneider D. S. & Soares M. P. Disease tolerance as a defense strategy. Science (New York, N.Y.) 335, 936–941 (2012). 10.1126/science.1214935 22363001 PMC3564547

[36] Raberg L., Sim D. & Read A. F. Disentangling genetic variation for resistance and tolerance to infectious diseases in animals. Science (New York, N.Y.) 318, 812–814 (2007). 10.1126/science.1148526 17975068

[37] Read A. F., Graham A. L. & Raberg L. Animal defenses against infectious agents: is damage control more important than pathogen control. PLoS Biol 6, e4 (2008). 10.1371/journal.pbio.1000004 PMC2605932 19222305

[38] Schneider D. S. & Ayres J. S. Two ways to survive infection: what resistance and tolerance can teach us about treating infectious diseases. Nat Rev Immunol 8, 889–895 (2008). 10.1038/nri2432 18927577 PMC4368196

[39] Raberg L., Graham A. L. & Read A. F. Decomposing health: tolerance and resistance to parasites in animals. Philos Trans R Soc Lond B Biol Sci 364, 37–49 (2009). 10.1098/rstb.2008.0184 18926971 PMC2666700

[40] Seixas E. Heme oxygenase-1 affords protection against noncerebral forms of severe malaria. Proc Natl Acad Sci U S A 106, 15837–15842 (2009). 10.1073/pnas.0903419106 19706490 PMC2728109

[41] Cohn O. Distinct gene programs underpinning disease tolerance and resistance in influenza virus infection. Cell Syst 13, 1002–1015 e1009 (2022). 10.1016/j.cels.2022.11.004 36516834

[42] Divangahi M., Khan N. & Kaufmann E. Beyond Killing Mycobacterium tuberculosis: Disease Tolerance. Front Immunol 9, 2976 (2018). 10.3389/fimmu.2018.02976 30619333 PMC6305711

[43] Downey J. Mitochondrial cyclophilin D promotes disease tolerance by licensing NK cell development and IL-22 production against influenza virus. Cell Rep 39, 110974 (2022). 10.1016/j.celrep.2022.110974 35732121

[44] Pernet E., Downey J., Vinh D. C., Powell W. S. & Divangahi M. Leukotriene B(4)-type I interferon axis regulates macrophage-mediated disease tolerance to influenza infection. Nature microbiology 4, 1389–1400 (2019). 10.1038/s41564-019-0444-3 31110361

[45] Ayres J. S. The Biology of Physiological Health. Cell 181, 250–269 (2020). 10.1016/j.cell.2020.03.036 32302569 PMC7409982

[46] Glaab T. Invasive versus noninvasive measurement of allergic and cholinergic airway responsiveness in mice. Respir Res 6, 139 (2005). 10.1186/1465-9921-6-139 16309547 PMC1316879

[47] Lomask M. Further exploration of the Penh parameter. Exp Toxicol Pathol 57 Suppl 2, 13–20 (2006). 10.1016/j.etp.2006.02.014 16638630

[48] Menachery V. D., Gralinski L. E., Baric R. S. & Ferris M. T. New Metrics for Evaluating Viral Respiratory Pathogenesis. PLoS One 10, e0131451 (2015). 10.1371/journal.pone.0131451 26115403 PMC4482571

[49] Palmer L. A. Hypoxia-induced ventilatory responses in conscious mice: gender differences in ventilatory roll-off and facilitation. Respir Physiol Neurobiol 185, 497–505 (2013). 10.1016/j.resp.2012.11.010 23183420 PMC3593587

[50] Winkler E. S. SARS-CoV-2 infection of human ACE2-transgenic mice causes severe lung inflammation and impaired function. Nature immunology 21, 1327–1335 (2020). 10.1038/s41590-020-0778-2 32839612 PMC7578095

[51] Daseke M. J. 2nd Exogenous IL-4 shuts off pro-inflammation in neutrophils while stimulating anti-inflammation in macrophages to induce neutrophil phagocytosis following myocardial infarction. Journal of molecular and cellular cardiology 145, 112–121 (2020). 10.1016/j.yjmcc.2020.06.006 32574573 PMC7483959

[52] Leonhardt J. Candida albicans beta-Glucan Differentiates Human Monocytes Into a Specific Subset of Macrophages. Front Immunol 9, 2818 (2018). 10.3389/fimmu.2018.02818 30555483 PMC6284042

[53] Allen J. E. IL-4 and IL-13: Regulators and Effectors of Wound Repair. Annual review of immunology 41, 229–254 (2023). 10.1146/annurev-immunol-101921-041206 36737597

[54] Chenery A. L. IL-13 deficiency exacerbates lung damage and impairs epithelial-derived type 2 molecules during nematode infection. Life Sci Alliance 4 (2021). 10.26508/lsa.202001000 PMC8321663 34127548

[55] Sutherland T. E. Ym1 induces RELMα and rescues IL-4Rα deficiency in lung repair during nematode infection. PLoS pathogens 14, e1007423 (2018). 10.1371/journal.ppat.1007423 30500858 PMC6291165

[56] Minutti C. M., Knipper J. A., Allen J. E. & Zaiss D. M. Tissue-specific contribution of macrophages to wound healing. Seminars in cell & developmental biology 61, 3–11 (2017). 10.1016/j.semcdb.2016.08.006 27521521

[57] Minutti C. M. Local amplifiers of IL-4Rα-mediated macrophage activation promote repair in lung and liver. Science (New York, N.Y.) 356, 1076–1080 (2017). 10.1126/science.aaj2067 28495878 PMC5737834

[58] Gause W. C., Wynn T. A. & Allen J. E. Type 2 immunity and wound healing: evolutionary refinement of adaptive immunity by helminths. Nat Rev Immunol 13, 607–614 (2013). 10.1038/nri3476 23827958 PMC3789590

[59] Lechner A. J. Recruited monocytes and type 2 immunity promote lung regeneration following pneumonectomy. 21, 120–134. e127 (2017). 10.1016/j.stem.2017.03.024 PMC5501755 28506464

[60] Thomas G. D. The biology of nematode- and IL4Rα-dependent murine macrophage polarization in vivo as defined by RNA-Seq and targeted lipidomics. Blood 120, e93–e104 (2012). 10.1182/blood-2012-07-442640 23074280 PMC4314526

[61] Impellizzieri D. IL-4 receptor engagement in human neutrophils impairs their migration and extracellular trap formation. The Journal of allergy and clinical immunology 144, 267–279.e264 (2019). 10.1016/j.jaci.2019.01.042 30768990

[62] Heeb L. E. M., Egholm C., Impellizzieri D., Ridder F. & Boyman O. Regulation of neutrophils in type 2 immune responses. Current opinion in immunology 54, 115–122 (2018). 10.1016/j.coi.2018.06.009 30015087

[63] Woytschak J. Type 2 Interleukin-4 Receptor Signaling in Neutrophils Antagonizes Their Expansion and Migration during Infection and Inflammation. Immunity 45, 172–184 (2016). 10.1016/j.immuni.2016.06.025 27438770

[64] Ajendra J. Lessons in type 2 immunity: Neutrophils in Helminth infections. Seminars in immunology 53, 101531 (2021). 10.1016/j.smim.2021.101531 34836773

[65] Ghoneim H. E., Thomas P. G. & McCullers J. A. Depletion of alveolar macrophages during influenza infection facilitates bacterial superinfections. J Immunol 191, 1250–1259 (2013). 10.4049/jimmunol.1300014 23804714 PMC4907362

[66] David C., Verney C., Si-Tahar M. & Guillon A. The deadly dance of alveolar macrophages and influenza virus. Eur Respir Rev 33 (2024). 10.1183/16000617.0132-2024 PMC11522969 39477353

[67] Hashimoto D. Tissue-resident macrophages self-maintain locally throughout adult life with minimal contribution from circulating monocytes. Immunity 38, 792–804 (2013). 10.1016/j.immuni.2013.04.004 23601688 PMC3853406

[68] Li F. Monocyte-derived alveolar macrophages autonomously determine severe outcome of respiratory viral infection. Sci Immunol 7, eabj5761 (2022). 10.1126/sciimmunol.abj5761 35776802

[69] Narasaraju T. Excessive neutrophils and neutrophil extracellular traps contribute to acute lung injury of influenza pneumonitis. The American journal of pathology 179, 199–210 (2011). 10.1016/j.ajpath.2011.03.013 21703402 PMC3123873

[70] Stoimenou M., Tzoros G., Skendros P. & Chrysanthopoulou A. Methods for the Assessment of NET Formation: From Neutrophil Biology to Translational Research. Int J Mol Sci 23 (2022). 10.3390/ijms232415823 PMC9781911 36555464

[71] Zhu S. Neutrophil extracellular traps contribute to immunothrombosis formation via the STING pathway in sepsis-associated lung injury. Cell Death Discov 9, 315 (2023). 10.1038/s41420-023-01614-8 37626060 PMC10457383

[72] Wang J. Bacterial colonization dampens influenza-mediated acute lung injury via induction of M2 alveolar macrophages. Nat Commun 4, 2106 (2013). 10.1038/ncomms3106 23820884 PMC3715851

[73] Islam M. M. & Takeyama N. Role of Neutrophil Extracellular Traps in Health and Disease Pathophysiology: Recent Insights and Advances. Int J Mol Sci 24 (2023). 10.3390/ijms242115805 PMC10649138 37958788

[74] Veras F. P. Targeting neutrophils extracellular traps (NETs) reduces multiple organ injury in a COVID-19 mouse model. Respir Res 24, 66 (2023). 10.1186/s12931-023-02336-2 36864506 PMC9978286

[75] Clevers H. The intestinal crypt, a prototype stem cell compartment. Cell 154, 274–284 (2013). 10.1016/j.cell.2013.07.004 23870119

[76] Hewitt R. J. Lung extracellular matrix modulates KRT5(+) basal cell activity in pulmonary fibrosis. Nat Commun 14, 6039 (2023). 10.1038/s41467-023-41621-y 37758700 PMC10533905

[77] Vaughan A. E. Lineage-negative progenitors mobilize to regenerate lung epithelium after major injury. Nature 517, 621–625 (2015). 10.1038/nature14112 25533958 PMC4312207

[78] Wei X. Macrophage peroxisomes guide alveolar regeneration and limit SARS-CoV-2 tissue sequelae. Science (New York, N.Y.) 387, eadq2509 (2025). 10.1126/science.adq2509 40048515 PMC12681967

[79] Glasser S. W. Surfactant protein C-deficient mice are susceptible to respiratory syncytial virus infection. Am J Physiol Lung Cell Mol Physiol 297, L64–72 (2009). 10.1152/ajplung.90640.2008 19304906 PMC2711816

[80] Ibanez L. I. Decreased expression of surfactant Protein-C and CD74 in alveolar epithelial cells during influenza virus A(H1N1)pdm09 and H3N2 infection. Microb Pathog 176, 106017 (2023). 10.1016/j.micpath.2023.106017 36736545

[81] Major J. Type I and III interferons disrupt lung epithelial repair during recovery from viral infection. Science (New York, N.Y.) 369, 712–717 (2020). 10.1126/science.abc2061 32527928 PMC7292500

[82] Kaufmann E. BCG Educates Hematopoietic Stem Cells to Generate Protective Innate Immunity against Tuberculosis. Cell 172, 176–190.e119 (2018). 10.1016/j.cell.2017.12.031 29328912

[83] Gurtner A., Crepaz D. & Arnold I. C. Emerging functions of tissue-resident eosinophils. The Journal of experimental medicine 220 (2023). 10.1084/jem.20221435 PMC10276195 37326974

[84] Mesnil C. Lung-resident eosinophils represent a distinct regulatory eosinophil subset. J Clin Invest 126, 3279–3295 (2016). 10.1172/JCI85664 27548519 PMC5004964

[85] Hu Y. Temporal and spatial atlas of eosinophil specialization across tissues. Nature immunology (2026). 10.1038/s41590-025-02382-6 41514064

[86] Filippone R. T. Potent CCR3 Receptor Antagonist, SB328437, Suppresses Colonic Eosinophil Chemotaxis and Inflammation in the Winnie Murine Model of Spontaneous Chronic Colitis. Int J Mol Sci 23 (2022). 10.3390/ijms23147780 PMC9317166 35887133

[87] Immler R. CCR3-dependent eosinophil recruitment is regulated by sialyltransferase ST3Gal-IV. Proc Natl Acad Sci U S A 121, e2319057121 (2024). 10.1073/pnas.2319057121 38687790 PMC11087806

[88] Yuan J. Gene knockdown of CCR3 reduces eosinophilic inflammation and the Th2 immune response by inhibiting the PI3K/AKT pathway in allergic rhinitis mice. Sci Rep 12, 5411 (2022). 10.1038/s41598-022-09467-4 35354939 PMC8969185

[89] Chen F. Helminth resistance is mediated by differential activation of recruited monocyte-derived alveolar macrophages and arginine depletion. Cell Rep 38, 110215 (2022). 10.1016/j.celrep.2021.110215 35021079 PMC9403845

[90] Reece J. J., Siracusa M. C. & Scott A. L. Innate immune responses to lung-stage helminth infection induce alternatively activated alveolar macrophages. Infect Immun 74, 4970–4981 (2006). 10.1128/IAI.00687-06 16926388 PMC1594865

[91] Sutherland T. E. Chitinase-like proteins promote IL-17-mediated neutrophilia in a tradeoff between nematode killing and host damage. Nature immunology 15, 1116–1125 (2014). 10.1038/ni.3023 25326751 PMC4338525

[92] Yue B. SPP1 induces idiopathic pulmonary fibrosis and NSCLC progression via the PI3K/Akt/mTOR pathway. Respir Res 25, 362 (2024). 10.1186/s12931-024-02989-7 39369217 PMC11456247

[93] Chen F. B Cells Produce the Tissue-Protective Protein RELMalpha during Helminth Infection, which Inhibits IL-17 Expression and Limits Emphysema. Cell Rep 25, 2775–2783 e2773 (2018). 10.1016/j.celrep.2018.11.038 30517865 PMC9413029

[94] Sutherland T. E. Ym1 induces RELMalpha and rescues IL-4Ralpha deficiency in lung repair during nematode infection. PLoS pathogens 14, e1007423 (2018). 10.1371/journal.ppat.1007423 30500858 PMC6291165

[95] Kotwica-Mojzych K., Jodlowska-Jedrych B. & Mojzych M. CD200:CD200R Interactions and Their Importance in Immunoregulation. Int J Mol Sci 22 (2021). 10.3390/ijms22041602 PMC7915401 33562512

[96] Minas K. & Liversidge J. Is the CD200/CD200 receptor interaction more than just a myeloid cell inhibitory signal? Critical reviews in immunology 26, 213–230 (2006). 10.1615/critrevimmunol.v26.i3.20 16928187 PMC2446434

[97] Snelgrove R. J. A critical function for CD200 in lung immune homeostasis and the severity of influenza infection. Nature immunology 9, 1074–1083 (2008). 10.1038/ni.1637 18660812

[98] Xie C. Macrophage Immunometabolism in Pulmonary Homeostasis and Chronic Lung Diseases. Int J Biol Sci 21, 6580–6598 (2025). 10.7150/ijbs.123492 41281740 PMC12631069

[99] Herold S., Mayer K. & Lohmeyer J. Acute lung injury: how macrophages orchestrate resolution of inflammation and tissue repair. Front Immunol 2, 65 (2011). 10.3389/fimmu.2011.00065 22566854 PMC3342347

[100] Zhao Q. Metabolic modeling of single bronchoalveolar macrophages reveals regulators of hyperinflammation in COVID-19. iScience 25, 105319 (2022). 10.1016/j.isci.2022.105319 36246577 PMC9549388

[101] Cai W. STAT6/Arg1 promotes microglia/macrophage efferocytosis and inflammation resolution in stroke mice. JCI Insight 4 (2019). 10.1172/jci.insight.131355 PMC6824303 31619589

[102] Lee Y. J. STAT6 Signaling Mediates PPARgamma Activation and Resolution of Acute Sterile Inflammation in Mice. Cells 10 (2021). 10.3390/cells10030501 PMC7996818 33652833

[103] Baker A. D. PPARgamma regulates the expression of cholesterol metabolism genes in alveolar macrophages. Biochem Biophys Res Commun 393, 682–687 (2010). 10.1016/j.bbrc.2010.02.056 20170635

[104] Rymut N. Resolvin D1 promotes efferocytosis in aging by limiting senescent cell-induced MerTK cleavage. FASEB J 34, 597–609 (2020). 10.1096/fj.201902126R 31914705 PMC6956736

[105] Huang S. Macrophage PPAR-gamma suppresses long-term lung fibrotic sequelae following acute influenza infection. PLoS One 14, e0223430 (2019). 10.1371/journal.pone.0223430 31584978 PMC6777801

[106] Adams T. S. Single-cell RNA-seq reveals ectopic and aberrant lung-resident cell populations in idiopathic pulmonary fibrosis. Sci Adv 6, eaba1983 (2020). 10.1126/sciadv.aba1983 32832599 PMC7439502

[107] Bosurgi L. Macrophage function in tissue repair and remodeling requires IL-4 or IL-13 with apoptotic cells. Science (New York, N.Y.) 356, 1072–1076 (2017). 10.1126/science.aai8132 28495875 PMC5556699

[108] Daniel B. The Nuclear Receptor PPARgamma Controls Progressive Macrophage Polarization as a Ligand-Insensitive Epigenomic Ratchet of Transcriptional Memory. Immunity 49, 615–626 e616 (2018). 10.1016/j.immuni.2018.09.005 30332629 PMC6197058

[109] Ferreira A. V. Fatty acid desaturation and lipoxygenase pathways support trained immunity. Nat Commun 14, 7385 (2023). 10.1038/s41467-023-43315-x 37968313 PMC10651900

[110] Xu-Vanpala S. Functional heterogeneity of alveolar macrophage population based on expression of CXCL2. Sci Immunol 5 (2020). 10.1126/sciimmunol.aba7350 PMC7717592 32769172

[111] Radtke A. J. IBEX: A versatile multiplex optical imaging approach for deep phenotyping and spatial analysis of cells in complex tissues. Proc Natl Acad Sci U S A 117, 33455–33465 (2020). 10.1073/pnas.2018488117 33376221 PMC7776876

[112] Pesce J. T. Arginase-1-expressing macrophages suppress Th2 cytokine-driven inflammation and fibrosis. PLoS pathogens 5, e1000371 (2009). 10.1371/journal.ppat.1000371 19360123 PMC2660425

[113] Morris S. M. Jr. Arginine metabolism: boundaries of our knowledge. J Nutr 137, 1602S–1609S (2007). 10.1093/jn/137.6.1602S 17513435

[114] Choi B. S. Differential impact of L-arginine deprivation on the activation and effector functions of T cells and macrophages. Journal of leukocyte biology 85, 268–277 (2009). 10.1189/jlb.0508310 19008294 PMC2642643

[115] Al-Kuraishy H. M. The Potential Nexus between Helminths and SARS-CoV-2 Infection: A Literature Review. J Immunol Res 2023, 5544819 (2023). 10.1155/2023/5544819 37383608 PMC10299886

[116] Naidoo P. SARS-CoV-2 and helminth co-infections, and environmental pollution exposure: An epidemiological and immunological perspective. Environ Int 156, 106695 (2021). 10.1016/j.envint.2021.106695 34171587 PMC8205275

[117] Adjobimey T., Meyer J., Terkes V., Parcina M. & Hoerauf A. Helminth antigens differentially modulate the activation of CD4(+) and CD8(+) T lymphocytes of convalescent COVID-19 patients in vitro. BMC Med 20, 241 (2022). 10.1186/s12916-022-02441-x 35764965 PMC9241220

[118] Merad M. & Martin J. C. Author Correction: Pathological inflammation in patients with COVID-19: a key role for monocytes and macrophages. Nat Rev Immunol 20, 448 (2020). 10.1038/s41577-020-0353-y PMC7201395 32376901

[119] Mueller A. L., McNamara M. S. & Sinclair D. A. Why does COVID-19 disproportionately affect older people? Aging 12, 9959–9981 (2020). 10.18632/aging.103344 32470948 PMC7288963

[120] Geckin B., Konstantin Föhse F., Domínguez-Andrés J. & Netea M. G. Trained immunity: implications for vaccination. Current opinion in immunology 77, 102190 (2022). 10.1016/j.coi.2022.102190 35597182

[121] Cirovic B. BCG Vaccination in Humans Elicits Trained Immunity via the Hematopoietic Progenitor Compartment. Cell Host Microbe 28, 322–334.e325 (2020). 10.1016/j.chom.2020.05.014 32544459 PMC7295478

[122] Quintin J. Candida albicans infection affords protection against reinfection via functional reprogramming of monocytes. Cell Host Microbe 12, 223–232 (2012). 10.1016/j.chom.2012.06.006 22901542 PMC3864037

[123] Shi C. & Pamer E. G. Monocyte recruitment during infection and inflammation. Nat Rev Immunol 11, 762–774 (2011). 10.1038/nri3070 21984070 PMC3947780

[124] Ziogas A. & Netea M. G. Trained immunity-related vaccines: innate immune memory and heterologous protection against infections. Trends in molecular medicine 28, 497–512 (2022). 10.1016/j.molmed.2022.03.009 35466062

[125] Zhang B. Single-cell RNA sequencing reveals induction of distinct trained-immunity programs in human monocytes. J Clin Invest 132 (2022). 10.1172/JCI147719 PMC8970681 35133977

[126] Desai P. Enteric helminth coinfection enhances host susceptibility to neurotropic flaviviruses via a tuft cell-IL-4 receptor signaling axis. Cell 184, 1214–1231 e1216 (2021). 10.1016/j.cell.2021.01.051 33636133 PMC7962748

[127] McFarlane A. J. Enteric helminth-induced type I interferon signaling protects against pulmonary virus infection through interaction with the microbiota. The Journal of allergy and clinical immunology 140, 1068–1078 e1066 (2017). 10.1016/j.jaci.2017.01.016 28196762 PMC6485385

[128] Reese T. A. Helminth infection reactivates latent gamma-herpesvirus via cytokine competition at a viral promoter. Science (New York, N.Y.) 345, 573–577 (2014). 10.1126/science.1254517 24968940 PMC4531374

[129] Zarek C. M. Preexisting helminth challenge exacerbates infection and reactivation of gammaherpesvirus in tissue resident macrophages. PLoS pathogens 19, e1011691 (2023). 10.1371/journal.ppat.1011691 37847677 PMC10581490

[130] Yao Y. Induction of Autonomous Memory Alveolar Macrophages Requires T Cell Help and Is Critical to Trained Immunity. Cell 175, 1634–1650 e1617 (2018). 10.1016/j.cell.2018.09.042 30433869

[131] Oyesola O. O. Exposure to lung-migrating helminth protects against murine SARS-CoV-2 infection through macrophage-dependent T cell activation. Sci Immunol 8, eadf8161 (2023). 10.1126/sciimmunol.adf8161 37566678

[132] Culemann S. Stunning of neutrophils accounts for the anti-inflammatory effects of clodronate liposomes. The Journal of experimental medicine 220 (2023). 10.1084/jem.20220525 PMC10067541 36976180

[133] Mass E. The stunning clodronate. The Journal of experimental medicine 220 (2023). 10.1084/jem.20230339 PMC10067525 36976179

[134] Piehler D. Eosinophils contribute to IL-4 production and shape the T-helper cytokine profile and inflammatory response in pulmonary cryptococcosis. The American journal of pathology 179, 733–744 (2011). 10.1016/j.ajpath.2011.04.025 21699881 PMC3157286

[135] Sabin E. A., Kopf M. A. & Pearce E. J. Schistosoma mansoni egg-induced early IL-4 production is dependent upon IL-5 and eosinophils. The Journal of experimental medicine 184, 1871–1878 (1996). 10.1084/jem.184.5.1871 8920874 PMC2192874

[136] Walsh E. R. Strain-specific requirement for eosinophils in the recruitment of T cells to the lung during the development of allergic asthma. The Journal of experimental medicine 205, 1285–1292 (2008). 10.1084/jem.20071836 18490489 PMC2413027

[137] Tiwary M., Rooney R. J., Liedmann S., LeMessurier K. S. & Samarasinghe A. E. Eosinophil Responses at the Airway Epithelial Barrier during the Early Phase of Influenza A Virus Infection in C57BL/6 Mice. Cells 10 (2021). 10.3390/cells10030509 PMC7997358 33673645

[138] Samarasinghe A. E. Eosinophils Promote Antiviral Immunity in Mice Infected with Influenza A Virus. J Immunol 198, 3214–3226 (2017). 10.4049/jimmunol.1600787 28283567 PMC5384374

[139] Matzinger P. The danger model: a renewed sense of self. Science (New York, N.Y.) 296, 301–305 (2002). 10.1126/science.1071059 11951032

[140] Caldarone L. Neutrophil extracellular traps in ex vivo lung perfusion perfusate predict the clinical outcome of lung transplant recipients. Eur Respir J 53 (2019). 10.1183/13993003.01736-2018 30655281

[141] Szturmowicz M. & Demkow U. Neutrophil Extracellular Traps (NETs) in Severe SARS-CoV-2 Lung Disease. Int J Mol Sci 22 (2021). 10.3390/ijms22168854 PMC8396177 34445556

[142] Veras F. P. SARS-CoV-2-triggered neutrophil extracellular traps mediate COVID-19 pathology. The Journal of experimental medicine 217 (2020). 10.1084/jem.20201129 PMC7488868 32926098

[143] Szanto A. STAT6 Transcription Factor Is a Facilitator of the Nuclear Receptor PPARγ-Regulated Gene Expression in Macrophages and Dendritic Cells. Immunity 33, 699–712 (2010). 10.1016/j.immuni.2010.11.009 21093321 PMC3052437

[144] Huang J. T. Interleukin-4-dependent production of PPAR-gamma ligands in macrophages by 12/15-lipoxygenase. Nature 400, 378–382 (1999). 10.1038/22572 10432118

[145] Ricote M., Welch J. S. & Glass C. K. Regulation of macrophage gene expression by the peroxisome proliferator-activated receptor-gamma. Horm Res 54, 275–280 (2000). 10.1159/000053271 11595817

[146] Rosenberg H. F., Dyer K. D. & Foster P. S. Eosinophils: changing perspectives in health and disease. Nat Rev Immunol 13, 9–22 (2013). 10.1038/nri3341 23154224 PMC4357492

[147] Kim Y. S. CD200R(high) neutrophils with dysfunctional autophagy establish systemic immunosuppression by increasing regulatory T cells. Cellular & molecular immunology 21, 349–361 (2024). 10.1038/s41423-024-01136-y 38311677 PMC10978921

[148] Clement M. IFITM3 restricts virus-induced inflammatory cytokine production by limiting Nogo-B mediated TLR responses. Nat Commun 13, 5294 (2022). 10.1038/s41467-022-32587-4 36075894 PMC9454482

[149] Doni Jayavelu N. Type 2 inflammation reduces SARS-CoV-2 replication in the airway epithelium in allergic asthma through functional alteration of ciliated epithelial cells. The Journal of allergy and clinical immunology 152, 56–67 (2023). 10.1016/j.jaci.2023.03.021 37001649 PMC10052850

[150] Murphy T. R. Patients with allergic asthma have lower risk of severe COVID-19 outcomes than patients with nonallergic asthma. BMC Pulm Med 22, 418 (2022). 10.1186/s12890-022-02230-5 36376851 PMC9660106

[151] Skevaki C. SARS-CoV-2 infection and COVID-19 in asthmatics: a complex relationship. Nat Rev Immunol 21, 202–203 (2021). 10.1038/s41577-021-00516-z 33623123 PMC7901163

[152] Nguyen T. H. O., Rowntree L. C., Chua B. Y., Thwaites R. S. & Kedzierska K. Defining the balance between optimal immunity and immunopathology in influenza virus infection. Nat Rev Immunol 24, 720–735 (2024). 10.1038/s41577-024-01029-1 38698083

[153] Wu X. BATF promotes group 2 innate lymphoid cell-mediated lung tissue protection during acute respiratory virus infection. Sci Immunol 7, eabc9934 (2022). 10.1126/sciimmunol.abc9934 35030033 PMC9005262

[154] McPhee C., Yevdokimova K., Rogers L. & Kraft M. The SARS-CoV-2 pandemic and asthma: What we have learned and what is still unknown. The Journal of allergy and clinical immunology 152, 1376–1381 (2023). 10.1016/j.jaci.2023.09.005 37739069

[155] Sansone N. M. S., Valencise F. E., Bredariol R. F., Peixoto A. O. & Marson F. A. L. Profile of coronavirus disease enlightened asthma as a protective factor against death: An epidemiology study from Brazil during the pandemic. Front Med (Lausanne) 9, 953084 (2022). 10.3389/fmed.2022.953084 36523782 PMC9745079

[156] Kim J. H. Inhibition of matrix metalloproteinase-9 prevents neutrophilic inflammation in ventilator-induced lung injury. Am J Physiol Lung Cell Mol Physiol 291, L580–587 (2006). 10.1152/ajplung.00270.2005 16698855

[157] Kayamuro H. Identification of new candidates as mucosal vaccine adjuvant in TNF family cytokines. Adv Exp Med Biol 691, 299–304 (2011). 10.1007/978-1-4419-6612-4_31 21153334

[158] Levine J. H. Data-Driven Phenotypic Dissection of AML Reveals Progenitor-like Cells that Correlate with Prognosis. Cell 162, 184–197 (2015). 10.1016/j.cell.2015.05.047 26095251 PMC4508757

[159] Subramanian A. Gene set enrichment analysis: a knowledge-based approach for interpreting genome-wide expression profiles. Proc Natl Acad Sci U S A 102, 15545–15550 (2005). 10.1073/pnas.0506580102 16199517 PMC1239896

[160] Tang Y. Exploiting the CD200-CD200R immune checkpoint axis in multiple myeloma to enhance CAR T-cell therapy. Blood 143, 139–151 (2024). 10.1182/blood.2022018658 37616575 PMC10862366

